# Genomic Features of *Taiwanofungus gaoligongensis* and the Transcriptional Regulation of Secondary Metabolite Biosynthesis

**DOI:** 10.3390/jof10120826

**Published:** 2024-11-27

**Authors:** Yadong Zhang, Yi Wang, Xiaolong Yuan, Hongling Zhang, Yuan Zheng

**Affiliations:** 1 College of Forestry, Southwest Forestry University, Kunming 650224, China; 2 Yunnan Key Laboratory of Biodiversity of Gaoligong Mountain, Yunnan Academy of Forestry and Grass-Land, Kunming 650201, China; yuanxiaolong@yafg.ac.cn; 3 College of Biological and Food Engineering, Southwest Forestry University, Kunming 650224, China

**Keywords:** *T. gaoligongensis*, whole-genome sequence, secondary metabolite, biosynthesis gene cluster, transcriptome sequencing, transcriptional regulation

## Abstract

Fungal secondary metabolites (SMs) have broad applications in biomedicine, biocontrol, and the food industry. In this study, whole-genome sequencing and annotation of *Taiwanofungus gaoligongensis* were conducted, followed by comparative genomic analysis with 11 other species of Polyporales to examine genomic variations and secondary metabolite biosynthesis pathways. Additionally, transcriptome data were used to analyze the differential expression of polyketide synthase (PKS), terpene synthase (TPS) genes, and transcription factors (TFs) under different culture conditions. The results show that *T. gaoligongensis* differs from other fungal species in genome size (34.58 Mb) and GC content (50.72%). The antibiotics and Secondary Metabolites Analysis Shell (AntiSMASH) analysis reveals significant variation in the number of SM biosynthetic gene clusters (SMBGCs) across the 12 species (12–29), with *T. gaoligongensis* containing 25 SMBGCs: 4 PKS, 6 non-ribosomal peptide synthetase (NRPS), and 15 TPS clusters. The *TgPKS1* gene is hypothesized to be involved in the biosynthesis of orsellinic acid or its derivatives, while *TgPKS2* might catalyze the synthesis of 6-methylsalicylic acid (6MSA) and its derivatives. The *TgTRI5* genes are suggested to synthesize tetracyclic sesquiterpene type B trichothecene compounds, while *TgPentS* may be involved in the synthesis of δ-cadinol, β-copaene, and α-murolene analogs or derivatives. Comparative genomic analysis shows that the genome size of *T. gaoligongensis* is similar to that of *T. camphoratus*, with comparable SMs. Both species share four types of PKS domains and five distinct types of TPS. Additionally, *T. gaoligongensis* exhibits a high degree of similarity to *Laetiporus sulphureus*, despite belonging to a different genus within the same family. Transcriptome analysis reveals significant variation in the expression levels of PKS and TPS genes across different cultivation conditions. The *TgPKS1* and *TgPKS4* genes, along with nine *TgTFs*, are significantly upregulated under three solid culture conditions. In contrast, under three different liquid culture conditions, the *TgPKS3*, *TgTRI5-1*, and *TgTRI5-2* genes, along with twelve *TgTFs*, exhibit higher activity. Co-expression network analysis and *TgTFs* binding site prediction in the promoter regions of *TgPKS* and *TgTPS* genes suggest that *TgMYB9* and *TgFTD4* regulate *TgPKS4* expression. *TgHOX1*, *TgHSF2*, *TgHSF3*, and *TgZnF4* likely modulate *TgPKS3* transcriptional activity. *TgTRI5-1* and *TgTRI5-5* expression is likely regulated by *TgbZIP2* and *TgZnF15*, respectively. This study provides new insights into the regulatory mechanisms of SMs in *T. gaoligongensis* and offers potential strategies for enhancing the biosynthesis of target compounds through artificial intervention.

## 1. Introduction

Fungal secondary metabolites (SMs) are a crucial source of lead compounds for drug discovery. These metabolites display a wide variety of structural frameworks, including polyketides, non-ribosomal peptides, terpenes, and alkaloids [1]. Numerous fungal SMs exhibit potent biological activities, including the broad-spectrum antibiotic penicillin, the lipid-lowering drug lovastatin, and the anticancer agent paclitaxel, whose core structures are synthesized by polyketide synthases (PKSs) or terpene synthases (TPSs) [2,3].

PKSs are essential enzymes in the synthesis of polyketide compounds, catalyzing the sequential decarboxylation and repeated condensation of multiple acyl-CoA units to generate polyketides. PKSs are primarily classified into three types: Type I, Type II, and Type III [4], with fungal PKSs predominantly categorized as Type I. These enzymes typically include the following major domains: keto-synthase (KS), acyltransferase (AT), dehydratase (DH), methyltransferase (MeT), enoyl reductase (ER), and acyl carrier protein (ACP) [5,6]. In 1990, Beck et al. first identified a fungal Type I PKS gene, 6-methylsalicylic acid synthase (6MSAS), through immuno-screening of a genomic DNA expression library using polyclonal antibodies [7]. TPSs are crucial for the biosynthesis of various terpenes. Terpenoid compounds consist of multiple isoprene units and can be classified based on their number into hemiterpenes (C5), monoterpenes (C10), sesquiterpenes (C15), diterpenes (C20), sesterterpenes (C25), triterpenes (C30), tetraterpenes (C40), and poly-terpenes (n > C40) [8]. In the terpene biosynthesis pathway, isoprene units are activated and converted into dimethylallyl pyrophosphate (DMAPP) and isopentenyl pyrophosphate (IPP), the common precursors for all terpenes. One DMAPP molecule and varying numbers of IPP molecules are processed by phenyl-transferases to produce different terpene precursors [9]. These precursors are subsequently converted into diverse terpene skeletons through the action of various TPSs [10].

Because SMs are generally not essential for the growth and development of organisms, the gene clusters associated with their synthesis are often in a “silent” state [11]. Consequently, strategies to activate or efficiently express these biosynthetic gene clusters (BGCs) have become a focal point in the research and application of SMs. Transcription factors (TFs), which are DNA-binding proteins that regulate gene transcription, play a critical role in modulating transcription efficiency. Genetic manipulation of these factors is regarded as a key approach for discovering novel SMs, as they can regulate the synthesis of SMs mediated by TPS or PKS gene clusters [12]. Fungal TFs can be categorized into two types based on their functional characteristics: pathway-specific TFs, which exhibit selective transcriptional regulation affecting only the BGCs within their coding sequences, and global TFs, which respond to external environmental signals, such as light, pH, temperature, and nutrients, thus regulating multiple BGCs either directly or indirectly [13].

With the rapid advancement of whole-genome sequencing technologies and bioinformatics, mining specific functional genes or gene clusters from genomes has become increasingly efficient. These technologies are invaluable for studying the biological functions of genes, providing reliable molecular evidence for genetics, secondary metabolism, biosynthetic pathways, and pathogen–host interactions in large wood-decaying fungi [14]. From the early days of Sanger sequencing to current high-throughput sequencing technologies, such as next-generation sequencing (NGS) and third-generation sequencing (TGS), genome mining has been widely applied in fields such as novel compound identification and metabolic engineering [15]. To date, the genomes of several valuable medicinal and edible fungi, including *Taiwanofungus camphoratus* [16,17], *Ganoderma lucidum* [18,19], *Inonotus obliquus* [20], and *Sanghuangporus sanghuang* [19], have been decoded, further facilitating the development of their medicinal value and industrial applications.

*Taiwanofungus* is a newly established genus based on molecular evidence that distinguishes it from the genus *Antrodia* in 2004 [21]. Currently, the genus *Taiwanofungus* includes *T. camphoratus*, *T. salmoneus*, and *T. gaoligongensis* [22]. Research on this genus has primarily focused on *T. camphoratus* (also known as *Antrodia camphorata* and *A. cinnamomea*). *T. camphoratus* contains various bioactive compounds, including polysaccharides, triterpenes, malic and succinic acid derivatives, superoxide dismutase, and sterols. These compounds exhibit a variety of biological functions. For instance, triterpenes have demonstrated hepatoprotective [23], antitumor [24,25], and antimicrobial properties [26]. Additionally, polysaccharides, as the primary metabolites of *T. camphoratus*, significantly affect blood sugar and lipid regulation [27] and enhance immune function [28]. Lu et al. revealed through genomic analysis various biosynthetic pathways for SMs in *T. camphoratus*, including sesquiterpenes, triterpenes, ergostane derivatives, andro-quinone, and andrographolide [16]. Yang et al. demonstrated that genes involved in SM synthesis in *T. camphoratus* are preferentially expressed in specific tissues, leading to the production of tissue-specific compounds [29]. Chen et al. isolated four monokaryotic strains from a dikaryotic strain of *T. camphoratus* and obtained high-quality genome sequences, revealing that *T. camphoratus* possesses a tetrapolar mating system. Compared to other edible fungi, *T. camphoratus* exhibits a notable reduction in gene families and individual gene counts, particularly those related to cell wall-degrading enzymes from plants, fungi, and bacteria. This reduction explains the rarity of *T. camphoratus* in natural environments, the difficulties and time-consuming nature of artificial cultivation, and its susceptibility to infections by other fungi and bacteria [17].

In this study, the whole genome of the *T. gaoligongensis* strain YAF008 was sequenced, functionally annotated, and analyzed for SM production using a combination of next-generation Illumina NovaSeq and third-generation Oxford Nanopore Technologies sequencing platforms. A BLAST domain analysis was performed, followed by the selection of 11 other species of Polyporales exhibiting over 97% similarity to at least one of the four PKS sequences of *T. gaoligongensis* for further comparative analysis. Based on these analyses, secondary metabolism-related PKS and TPS gene clusters in *T. gaoligongensis* were further explored. Additionally, using the whole-genome data of *T. gaoligongensis*, TFs from 10 families—Myeloblastosis (MYB), Basic Helix-Loop-Helix (bHLH), Basic Leucine Zipper (bZIP), Homeobox (HOX), Fungal Transcription Factor Domain (FTD), Winged Helix-Turn-Helix (WHTH), Zinc Finger (ZnF), Transcription Factor IIB (TFIIB), Heat Shock Factor (HSF), and High Mobility Group (HMG)—were identified and subjected to bioinformatics analysis. Transcriptome sequencing was employed to examine the expression of TgTPS, TgPKS, and TgTFs under various cultivation conditions and to investigate the TFs regulating the expression of TPS and PKS gene clusters. This research provides valuable insights into the exploration of SM genes and their transcriptional regulation in *Taiwanofungus* fungi.

## 2. Materials and Methods

### 2.1. Microbial Strains and Culture Conditions

The *T. gaoligongensis* strain was isolated in 2018 from the fallen bark of a camphor tree collected at Gaoligong Mountain, Yunnan, China. After cleaning, grinding, and homogenizing the bark, the diluted homogenate was plated on malt/yeast medium supplemented with antibiotics and incubated at 28 °C for 15 days to isolate a single colony. The strain was identified as *T. gaoligongensis* based on ITS sequencing, and the ITS sequence was deposited in GenBank under accession number OR681872. It has been deposited in the Yunnan Key Laboratory of Bio-diversity of Gaoligong Mountain, Yunnan Academy of Forestry and Grassland Sciences in Kunming, and the China Center for Type Culture Collection (deposit number: CCTCC M 20232425) in Wuhan, China. The strain was inoculated onto Potato Dextrose Agar and cultured in a constant temperature incubator at 26 °C with 60% humidity for 15 days before storage at 4 °C. For experimental procedures, 0.5 cm² of mycelium was uniformly excised from the edge of *T. gaoligongensis* mycelia and inoculated into various media, incubating at 28 °C for 15 days.

### 2.2. Preparation of Culture Media

The medium for genomic sequencing was malt/yeast extract medium.

For transcriptome sequencing, the liquid culture medium was prepared with the following composition, T: pea powder (5 g/L), KH_2_PO_4_ (1 g/L), MgSO_4_ (0.5 g/L), yeast powder (5 g/L), and vitamin B1 (0.1 g/L). Variants included: NFT (100 μL Triton X-100 + 5 g/L Cinnamomum kanehirae sawdust) and YFT (100 μL Triton X-100 + 5 g/L C. burmannii sawdust). The formula for the solid culture medium consisted of 15 mL MM medium, which contained NaNO_3_ (6 g/L), KCl (0.52 g/L), MgSO_4_ (0.52 g/L), and KH_2_PO_4_ (1.52 g/L). Variants included: YY (4 g Populus alba sawdust), YM (4 g Zea mays flour), and YR (4 g Coix Coicis Semenurr). All media were autoclaved at 121 °C for 20 min.

### 2.3. Genome Sequencing and Assembly

After 15 days of liquid culture, samples of *T. gaoligongensis* were collected for high-throughput sequencing at Shanghai Personalbio Biotechnology Co., Ltd., Shanghai, China. The whole genome shotgun approach was employed, utilizing 400 bp insert fragments to construct the gene library of *T. gaoligongensis*. Sequencing was conducted using NGS technology on the Illumina NovaSeq platform, along with TGS technology. Raw data were processed using FastQC. The 3′-terminal DNA junctions were decontaminated using AdapterRemoval (version 2) [30], and quality correction of all reads was performed using Soapec (V2.0) software. The KMER frequency for correction was set to 17 to obtain high-quality adaptor-free genome sequences. The data were assembled de novo to construct contigs and scaffolds. The obtained contigs and scaffolds were corrected using Pilon v1.18 [31] software.

### 2.4. Gene Prediction and Annotation

Augustus (version 3.03), GlimmerHMM (version 3.0.1), and GeneMark-ES (version 4.35) were used for de novo gene model prediction of this genome [32,33,34], yielding the corresponding gene prediction results. Homologous gene prediction was conducted using Exonerate (version 2.2.0) with protein sequences from closely related species. Finally, EVidenceModeler (version r2012-06-25) was used to integrate the de novo predictions with homologous predictions from related species [35]. The functions of the gene products were annotated based on BLAST searches against non-redundant protein sequences from NCBI (Nr), Swiss-Prot, Kyoto Encyclopedia of Genes and Genomes (KEGG), Evolutionary Genealogy of Genes: Non-supervised Orthologous Groups (EggNOG), Pathogen–Host Interactions Database (PHI), and Carbohydrate-Active Enzymes (CAZy).

### 2.5. Secondary Metabolite Biosynthesis Gene Cluster Analysis

Whole genome data for *T. camphoratus* 1, *T. camphoratus* 2, *D. quercina*, *W. cocos*, *L. sulphureus*, *F. radiculosa*, *F. palustris*, *F. schrenkii*, *F. betulina*, *P. placenta*, and *N. serialis* were sourced from NCBI (https://blast.ncbi.nlm.nih.gov/, accessed on 17 July 2024). The login IDs and strain IDs are listed in Table 1 The antiSMASH online tool (https://antismash.secondarymetabolites.org/, accessed on 17 July 2024) was used to predict gene clusters in the scaffolds of *T. gaoligongensis* and the genomes of the other 11 strains. Gene structure prediction was conducted using the FGENESH online tool (https://softberry.com/, accessed on 17 July 2024). The PKS/NRPS analysis tool (https://nrps.igs.umaryland.edu/, accessed on 17 July 2024) was used to predict gene clusters within contigs containing PKS genes and to identify domains. Additionally, Protein BLAST (https://blast.ncbi.nlm.nih.gov/, accessed on 17 July 2024) was used for protein alignment on these contigs.

### 2.6. Cluster Analysis

Known PKS and TPS protein sequences were downloaded from NCBI and compared with the protein sequences from this study using the Clustal W program in the MEGA 5.0 software of IQ-TREE. The IQ-TREE web server quickly and accurately generates phylogenetic trees using the maximum likelihood method (http://iqtree.cibiv.univie.ac.at/, accessed on 17 July 2024). The analysis involved 1000 bootstrap iterations using default parameters to construct the cluster tree.

### 2.7. Synteny Analysis

Scaffolds containing SMBGCs from the genomes of *T. gaoligongensis*, *T. camphoratus* 1, *T. camphoratus* 2, and *L. sulphureus* were analyzed for collinearity using MAUVE v2.4.0, following the assembly order.

### 2.8. Prediction of TPS Proteins

InterProScan v5.44-79.0 [36] was used to identify TPS proteins in *T. gaoligongensis* based on the associated terms for their conserved domains: Trichodiene synthase (TRI5, IPR024652), Pentalene synthase (Pents, IPR050225), geranylgeranyl pyrophosphate synthase (GGPPS: IPR000092), prenyltransferase (PTase, IPR039653), and squalene synthase (SQS, IPR006449). The candidate gene sequences obtained were compared against the NCBI protein database for confirmation. Subsequently, multiple sequence alignment was conducted using DNAMAN software version 6 to identify conserved domains.

### 2.9. Identification and Analysis of Transcription Factors

Domain files for MYB (PF00249), bHLH (PF00010), bZIP (PF00170), HOX (PF00046), FTD (PF04082), WHTH (PF00250), ZnF (PF16297), TFIIB (PF08613), HSF (PF00447), and HMG (PF00505) TFs were downloaded from the InterProScan database. HMMER software version 3.4 was used for global alignment and screening of protein sequences in the *T. gaoligongensis* genome, with a threshold set to an E value of <10^–5^. Short sequences (less than 100 amino acids) were manually excluded.

### 2.10. Transcriptome Sequencing and Differential Gene Expression Analysis

Utilizing NGS technology based on the Illumina HiSeq platform and employing paired-end sequencing methods, we conducted sequencing on samples grown under 6 different cultivation conditions (T: Pea powder (5 g/L), KH₂PO₄ (1 g/L), MgSO₄ (0.5 g/L), yeast powder (5 g/L), and vitamin B1 (0.1 g/L), NFT: T medium supplemented with 100 μL Triton X-100 and 5 g/L *C. kanehirae* sawdust, YFT: T medium supplemented with 100 μL Triton X-100 and 5 g/L *C. burmannii* sawdust, YY: 15 mL MM medium with 4 g Populus alba sawdust, YM: 15 mL MM medium with 4 g Zea mays flour, YR: 15 mL MM medium with 4 g Coix Coicis Semenurr). By referencing the sequence numbers of PKS, TPS and TFs from the genomic data, we quantified their gene expression levels in the transcriptome data as fragments per kilobase of transcript per million mapped reads (FPKM) values. We employed TBtools software version 2.056 to create interactive heatmaps for analyzing the expression levels of target genes.

### 2.11. Real-Time Quantitative Fluorescence PCR

Genes exhibiting similar expression patterns under various cultivation conditions were selected, including *TgTFs*, *TgPKS*, and *TgTPS* genes such as *TgHSF2*, *TgHSF3*, *TgHOX1*, *TgZnF4*, *TgZnF15*, *TgbZIP2*, *TgMYB9*, *TgFTD4*, *TgPKS3*, *TgPKS4*, *TgTRI5-1* and *TgTRI5-5*. Primers for these genes were designed using Primer Premier 5.0 software to evaluate their expression levels (Table S1). The PCR reaction mixture consisted of 20 µL total volume, comprising 10 µL of PCR mix, 1 µL of DNA/cDNA template, 2 µL of primers, and 7 µL of deionized water. The PCR conditions included an initial denaturation at 94 °C for 2 min, followed by 40 amplification cycles (94 °C for 15 s, 65 °C for 15 s, 72 °C for 45 s), and a final extension at 72 °C for 10 min.

### 2.12. Prediction of Transcription Factor Binding Sites

Using the whole-genome and transcriptome data of *T. gaoligongensis*, DNA sequences 2000 bp upstream of the start codon of PKS and TPS genes that exhibit similar expression patterns to TFs were extracted with TBtools software version 2.056. Potential binding sites for TFs to their co-expressed gene promoter regions were subsequently predicted using the JASPAR online tool, with a confidence level set at 90% [37].

## 3. Results

### 3.1. Basic Features of the T. gaoligongensis Genome

#### 3.1.1. Genome Annotation

Illumina sequencing produced 36,080,652 high-quality reads, with a 99.39% HQReadsP. The genome of *T. gaoligongensis* is 34.58 Mb, consisting of 19 contigs and 19 scaffolds, with a scaffold N50 of 2,343,139 bp and a GC content of 50.72%. A total of 7955 protein-coding genes were predicted, with a cumulative length of 14.79 Mb, an average sequence length of 1858.7 bp, and the longest contig at 0.42 Mb (Table 2), tRNA genes were predicted using tRNAscan-SE (version 1.3.1) (Lowe TM and Eddy SR, 1997), rRNA genes with RNAmmer 1.2 [38], and other non-coding RNAs were identified mainly through Rfam comparison [39]. This analysis predicted 136 tRNA secondary structures, 51 rRNA genes, and 26 snRNA genes using tRNAscan, RNAmmer, and rfam_scan, respectively.

#### 3.1.2. Genome Annotation of *T. gaoligongensis*

**Figure 1:**
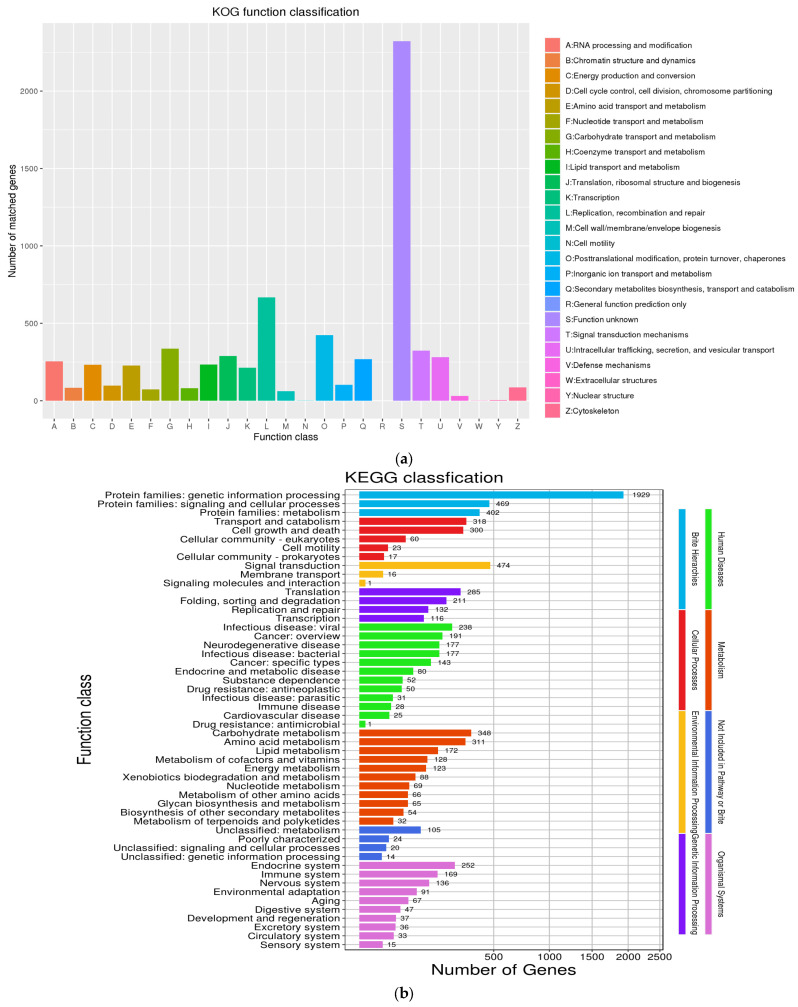

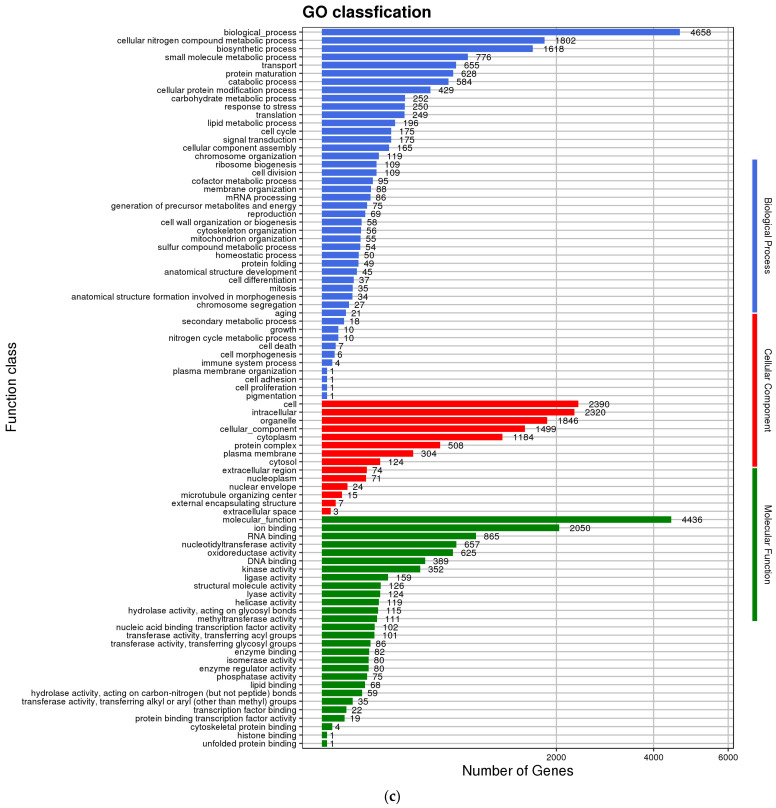
Functional annotation of *T. gaoligongensis* genes encoding the proteins: (**a**) eggNOG analysis; (**b**) KEGG analysis; (**c**) GO analysis.

The 7955 non-redundant genes predicted in the *T. gaoligongensis* genome were functionally annotated using several databases, including NR, SwissProt, KEGG, GO, eggNOG, and Pfam. A total of 7548, 5204, 2874, 5148, 6700, and 5613 genes were annotated in each database, respectively (Table 3). EggNOG annotation showed that the most abundant gene categories were “Function unknown” (2323), “Replication, recombination, and repair” (667), and “Posttranslational modification, protein turnover, chaperones” (424) (Figure 1a). KEGG enrichment analysis showed that the genes were mainly enriched in pathways for genetic information processing (1929), signal transduction (474), and cellular signaling and processing (469) (Figure 1b). This suggests a rich diversity of genetic and signaling proteins, potentially enhancing information exchange and secondary metabolism. GO annotation classified the genes into cellular components, molecular functions, and biological processes, with the highest numbers in the cell, biological process, and molecular function categories (Figure 1c). This indicates a large number of genes related to cellular components, biological processes, and molecular functions, which may help maintain cellular structure and metabolic efficiency, supporting the survival and adaptability of *T. gaoligongensis*. The *T. gaoligongensis* strain likely contains several metabolic genes involved in signal transduction.

#### 3.1.3. Additional Annotation of *T. gaoligongensis*

##### Pathogen–Host Interactions (PHI)

**Figure 2:**
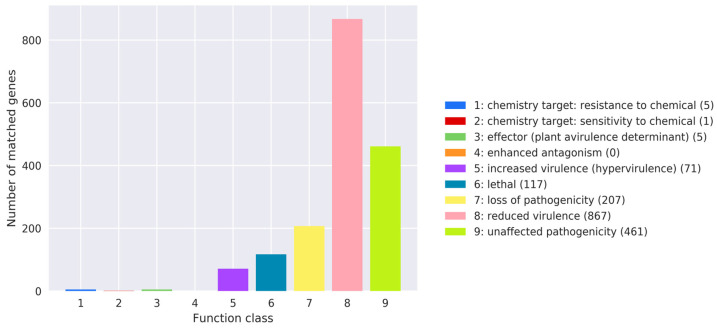
Distribution map of mutation types in the pathogen PHI phenotype of *T. gaoligongensis*.

The Pathogen–Host Interactions database (PHI-base) primarily includes pathogens from fungi, oomycetes, and bacteria, with hosts such as animals, plants, fungi, and insects [40]. Amino acid sequences of *T. gaoligongensis* were aligned using the PHI-base database to obtain annotation results (Figure 2). *T. gaoligongensis* harbors a diverse set of PHI-base genes, including reduced virulence (867), unaffected pathogenicity (461), loss of pathogenicity (207), lethal (117), increased virulence (hypervirulence) (71), effector (plant avirulence determinant) (5), resistance to chemicals (5), and sensitivity to chemicals (1). The predominant annotations are reduced virulence and unaffected pathogenicity, suggesting that *T. gaoligongensis* is not a highly pathogenic strain.

##### Carbohydrate Genes

**Figure 3:**
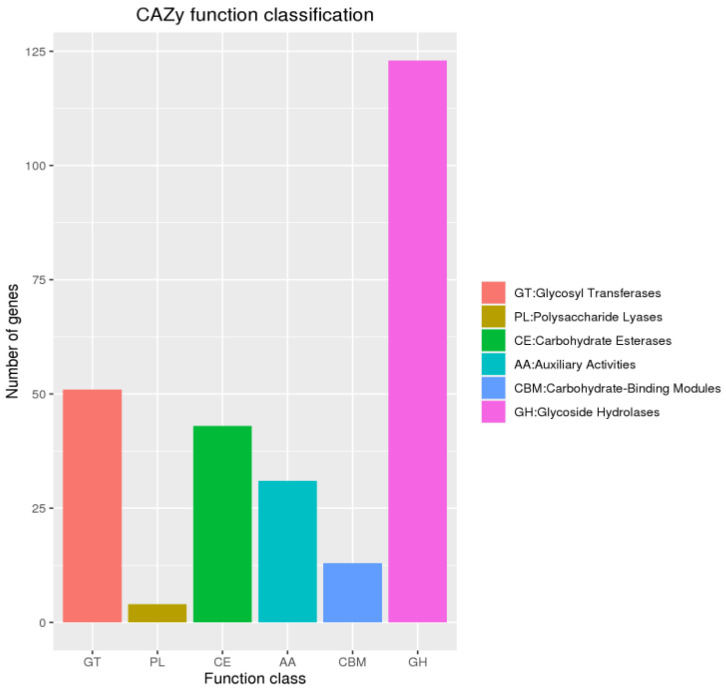
CAZy functional classification chart of *T. gaoligongensis*.

Analysis identified 265 CAZy genes in *T. gaoligongensis*, comprising 123 glycoside hydrolases (GHs), 51 glycosyl transferases (GTs), 43 carbohydrate esterases (CEs), 31 auxiliary activities (AAs), 13 carbohydrate-binding modules (CBMs), and 4 polysaccharide lyases (PLs) (Figure 3). CAZy enzymes catalyze the assembly and breakdown of glycans and glycoconjugates [41]. Additionally, CAZy was a database of carbohydrate-active and carbohydrate enz [42]. It is suggested that *T. gaoligongensis* may efficiently capture energy and degrade complex carbohydrates.

##### Transporter Classification Database

**Figure 4:**
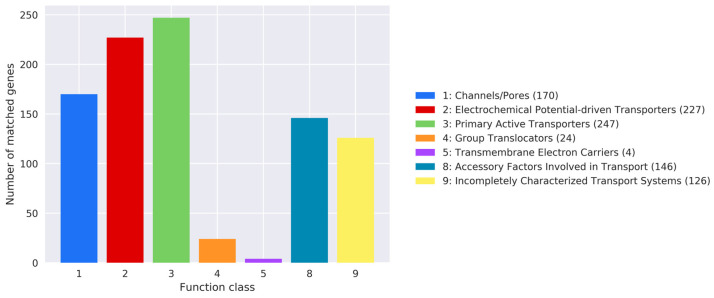
TCDB Functional Classification Chart of *T. gaoligongensis*.

The Transporter Classification Database (TCDB) is a freely accessible reference for transport protein research, offering structural, functional, mechanistic, evolutionary, and disease-related information on transporters from various organisms [43]. Analysis shows that the strain contains a diverse array of cell membrane transport proteins, including 247 Primary Active Transporters, 227 Electrochemical Potential-driven Transporters, 170 Channels/Pores, 146 Accessory Factors Involved in Transport, 126 Incompletely Characterized Transport Systems, 24 Group Translocators, and 4 Transmembrane Electron Carriers (Figure 4). This suggests a broad capacity and functional diversity for substance transport in this strain.

### 3.2. Genomic Characteristics of 12 Strains

The genome of *T. gaoligongensis* was analyzed and compared with 11 other fungal species. The genome of *N. serialis* is the largest (66.7 Mb), and *F. radiculosa* has the smallest genome (28.4 Mb). The genome size of *T. gaoligongensis* (34.58 Mb) is similar to that of its congeners *T. camphoratus1* (33 Mb) and *T. camphoratus2* (32.2 Mb). Due to differences in sequencing platforms and assembly techniques, the number of scaffolds varies significantly, with *N. serialis* having the most (893) and *T. camphoratus1* the fewest (14). *T. gaoligongensis* has 19 scaffolds. The GC content of the 12 genomes ranges from 50.5% to 56% (Table 1).

### 3.3. Analysis of Secondary Metabolite Biosynthesis Gene Cluster

SM biosynthesis genes are typically organized into BGCs within the genome. AntiSMASH analysis reveals that *N. serialis* contains the highest number of SM BGCs (29), while *F. palustris* has the lowest (12). Both *T. gaoligongensis* and *T. camphoratus1* contain 25 SM BGCs, while *T. camphoratus2* has 23. *N. serialis* also has the most PKS genes (9), followed by *F. schrenkii* and *F. radiculosa* (6). Additionally, *N. serialis* possesses the most NRPS genes (7), whereas *F. schrenkii* and *F. radiculosa* each have only 1. In the Taiwanofungus genus, both *T. gaoligongensis* and *T. camphoratus1* have an equal number of PKS genes (4) and TPS genes (15). Meanwhile, *T. camphoratus2* has 4 NRPS genes, while both *T. gaoligongensis* and *T. camphoratus1* possess 6 NRPS genes (Table 3). This indicates variation in the number and types of SM BGCs within and between genera in the same family.

**Figure 5:**
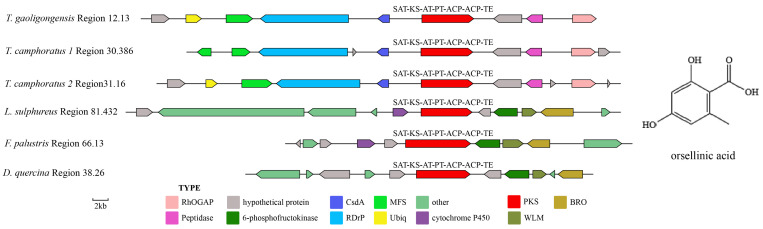
Comparison of biosynthesis of putative orsellinic acid biosynthetic gene clusters. The number after the region and the number before the decimal point represent the scaffold, and the number after the decimal point represents the gene cluster.

*T. gaoligongensis* contains 4 PKS genes: 1 NR-PKS, 2 PR-PKS, and 1 HR-PKS, characterized by the following domain structures: *TgPKS1* (SAT-KS-AT-PT-ACP-ACP-TE), *TgPKS2* (KS-AT-DH-KR-ACP-TE), *TgPKS3* (KS-AT-KR-ACP-TE), and *TgPKS4* (KS-AT-DH-MT-ER-KR-ACP-TE). Among the 11 genomes compared, five proteins similar to TGPKS1 were identified: *T. camphoratus1* Region30.386, *T. camphoratus2* Region31.16, *L. sulphureus* Region81.432, *F. palustris* Region66.13, and *D. quercina* Region38.26, all possessing the domain composition SAT-KS-AT-PT-ACP-ACP-TE. Phylogenetic analysis shows that these six genes cluster closely with the NR-PKS of *Aspergillus nidulans*, exhibiting 87–100% similarity (Figure S1). The NR-PKS of *A. nidulans* primarily produces orsellinic acid [44], suggesting that TGPKS1 is likely involved in the synthesis of orsellinic acid or its derivatives. The NR-PKS gene clusters in the genus *Taiwanofungus* contain RhOGAP, CsDA, MFS, RDrP, and Peptidase genes, along with instances of gene loss and horizontal gene transfer. Additionally, the modifying genes in *L. sulphureus* Region81.432, *F. palustris* Region66.13, and *D. quercina* Region38.26 contain WLM, BRO1, and 6-phosphofructokinase genes, which are absent in *Taiwanofungus* species (Figure 5). This demonstrates the high conservation of core PKS genes involved in orsellinic acid synthesis across different species, along with significant diversity in the surrounding modifying genes, indicating a coexistence of high conservation and diversity in the biosynthetic pathways.

**Figure 6:**
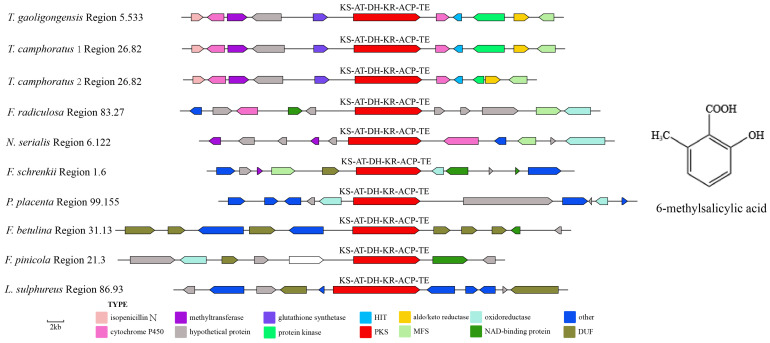
Comparison of biosynthesis of putative 6MSA biosynthetic gene clusters. The number after the region and the number before the decimal point represent the scaffold, and the number after the decimal point represents the gene cluster.

In the genomes of *T. camphoratus1*, *T. camphoratus2*, *F. radiculosa*, *N. serialis*, *F. schrenkii*, *P. placenta*, *F. betulina*, *F. pinicola*, and *L. sulphureus*, proteins similar to TgPKS2 were identified, all containing a PR-PKS with the domain structure KS-AT-DH-KR-ACP-TE. A cluster analysis of these nine PR-PKS protein sequences, TgPKS2, and three PKS genes related to 6MSA synthesis obtained from NCBI revealed that these 10 PR-PKS genes share up to 96% homology with the PKS genes involved in 6MSA synthesis, suggesting that TgPKS2 may catalyze the synthesis of 6MSA or its derivatives (Figure S2). The modification genes in *T. gaoligongensis* Region5.533, *T. camphoratus1* Region26.82, and *T. camphoratus2* Region14.77 are identical, all containing isopenicillin N, cytochrome P450, methyltransferase, glutathione synthetase, HIT, protein kinase, aido/keto reductase, and MFS. Additionally, the BGCs in *F. radiculosa* Region83.27, *F. schrenkii* Region1.6, *P. placenta* Region99.155, and *F. pinicola* Region21.3 all include NAD-binding proteins and oxidoreductases (Figure 6).

**Figure 7:**
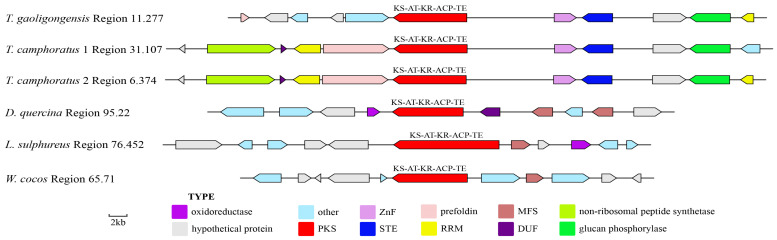
Comparative Analysis of Genes Surrounding *TgPKS3* in *T. gaoligongensis* and Related Species. The number after the region and the number before the decimal point represent the scaffold, and the number after the decimal point represents the gene cluster.

TgPKS3 shares structural similarity with TgPKS2 but lacks a DH domain. The analysis revealed that *T. camphoratus1* Region31.107, *T. camphoratus2* Region6.374, *D. quercina* Region95.22, *L. sulphureus* Region76.452, and *W. cocos* Region65.71 contain proteins similar to TgPKS3, all exhibiting the domain structure KS-AT-KR-ACP-TE. The gene clusters in *T. gaoligongensis* Region11.277, *T. camphoratus1* Region31.107, and *T. camphoratus2* Region6.374 all include RRM, alpha-glucan phosphorylase, ZnF, STE, and prefoldin. However, *T. gaoligongensis* lacks DUF and non-ribosomal peptide synthetase, and its prefoldin is noticeably shorter than that of the other two species. Significant differences exist in the modification genes of the other three BGCs, with only one shared MFS gene (Figure 7).

**Figure 8:**
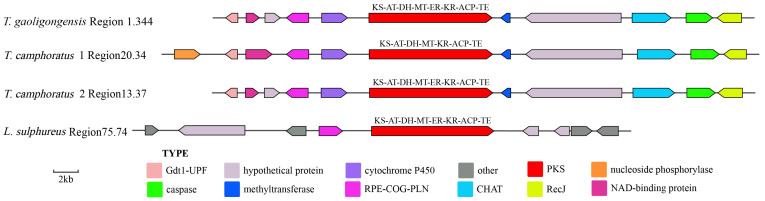
Comparative Analysis of Genes Surrounding *TgPKS4* and Related Species. The number after the region and the number before the decimal point represent the scaffold, and the number after the decimal point represents the gene cluster.

Only three proteins similar to TgPKS4 were identified, located in *T. camphoratus1* Region20.34, *T. camphoratus2* Region13.37, and *L. sulphureus* Region75.74, all sharing the domain composition KS-AT-DH-MT-ER-KR-ACP-TE. The modification genes in *T. gaoligongensis* Region1.34, *T. camphoratus1* Region20.34, and *T. camphoratus2* Region13.37 exhibit high similarity, all containing Gdt1-UPF, NAD-binding protein, REP-COG-PLN, methyltransferase, CHAT, caspase, and RecJ. In contrast, the modification genes in *L. sulphureus* Region75.74 differ significantly from those in fungi of the genus *Taiwanofungus* (Figure 8).

### 3.4. Synteny Analysis of Four Species of Polyporales

**Figure 9:**
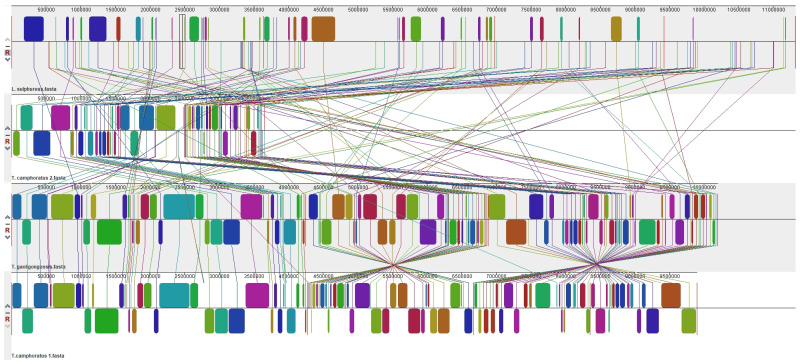
Scaffold containing SM biosynthesis gene cluster used for synteny analysis. From top to bottom: *L. sulphureus*, *T. camphoratus2*, *T. gaoligongensis*, *T. camphoratus1*.

A synteny analysis was conducted on the scaffolds containing PKS genes in the genomes of *T. gaoligongensis* (1, 5, 11, 12), *T. camphoratus1* (20, 26, 30, 31), *T. camphoratus2* (6, 13, 14, 31), and *L. sulphureus* (75, 76, 81, 86). The results revealed 112 homologous regions between *T. gaoligongensis* and the other three species. *T. gaoligongensis* and *T. camphoratus1* exhibited high levels of homology, with similar sizes and relative positions of the homologous regions. This suggests that these regions may be under conserved selection pressure and could play crucial biological roles in both species. In contrast, the homologous regions between *T. camphoratus2* and *L. sulphureus* showed significant differences in size and relative position, possibly due to genome rearrangements, independent evolutionary paths, or gene loss/gain events. Additionally, gene variations and rearrangements were relatively minor within species of the same genus, allowing for greater genome structure conservation. Conversely, inter-genus species exhibited more pronounced gene variation and rearrangement, reflecting lower synteny (Figure 9).

### 3.5. Characterization of TgTPS Proteins

**Figure 10:**
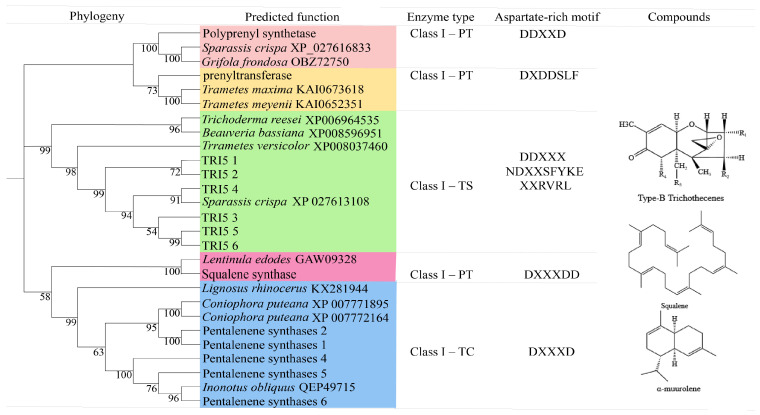
Genomic inventory for terpenoid biosynthesis in *T. gaoligongensis*.

A total of 15 TPS genes were identified in the genome of *T. gaoligongensis*, including 6 TRI5, 6 PentS, 1 GGPPS, 1 PTase, and 1 SQS (Figure 10). All TgTRI5 proteins contain Mg²⁺ binding sites (DDXXX), a pyrophosphate and ion chelation region (NDXXSFYKE), and an FPP-binding motif (XXRVRL) (Figure S3). Phylogenetic analysis indicates that the TgTRI5 protein sequences cluster with the TRI5 proteins from *Sparassis crispa* (XP_027613108), *Beauveria bassiana* (XP_008596951), *Trichoderma reesei* (XP_006964535), and *Trametes versicolor* (XP_008037460), all of which likely encode trichodiene synthases that produce tetracyclic sesquiterpene B-type trichodiene compounds. TgPentS contains the conserved “DXXXD” sequence and exhibits high homology with the sesquiterpene synthases of *Coniophora puteana* (XP_007771895; XP_007772164), *Lignosus rhinocerus* (KX281944), and *Inonotus obliquus* (QEP49715) (Figure S4), suggesting it may be involved in synthesizing δ-cadinol, β-copaene, or α-muurolene analogs or derivatives. TgGGPPS contains the conserved “DDXXD” sequence and shares 100% homology with the GGPPS from *Sparassis crispa* (XP_027616833) and *Grifola frondosa* (OBZ72750) (Figure S5). TgPTase contains the conserved “DXDDSLF” sequence and shows high homology with isoprenyl transferases from *Trametes maxima* (KAI0673618) and *Trametes meyenii* (KAI0652351) (Figure S6). Additionally, TgSQS (IPR006449), whose active site includes the “DXXXDD” motif, clusters with the SQS from *Lentinula edodes* (GAW09328) (Figure S7).

### 3.6. Construction and Comparative Analysis of the TPS Phylogenetic Tree

**Figure 11:**
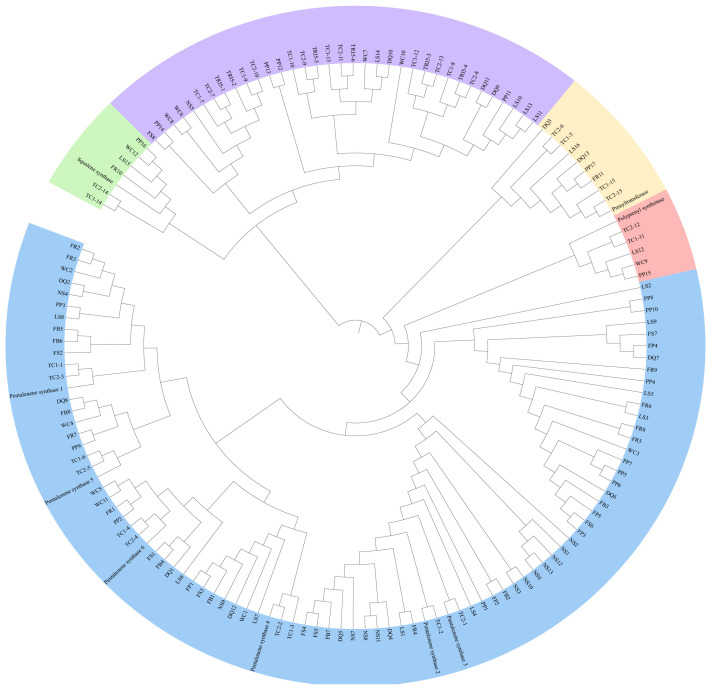
Phylogenetic Tree of TPS Proteins from 12 Fungal Strains. The TgTPS types are indicated in the figure: TC1 (*T. camphoratus1*), TC2 (*T. camphoratus2*), DQ (*D. quercina*), WC (*W. cocos*), LS (*L. sulphureus*), FR (*F. radiculosa*), FP (*F. palustris*), FS (*F. schrenkii*), FB (*F. betulina*), PP (*P. placenta*), NS (*N. serialis*).

A predictive analysis of the TRI5, PentS, GGPPS, PTase, and SQS genes across 12 fungal genomes identified a total of 148 TPS genes. The number of TPS genes in the fungi of the genus *Taiwanofungus* was consistent, with all species containing these five distinct types of TPS. However, *T. gaoligongensis* has one additional *TRI5* gene and one fewer *PentS* gene compared to *T. camphoratus1* and *T. camphoratus2*. Additionally, *L. sulphureus* also possesses all five types of TPS. The phylogenetic tree indicates that the 148 TPS genes are divided into five distinct clades based on their types (Figure 11).

**Figure 12:**
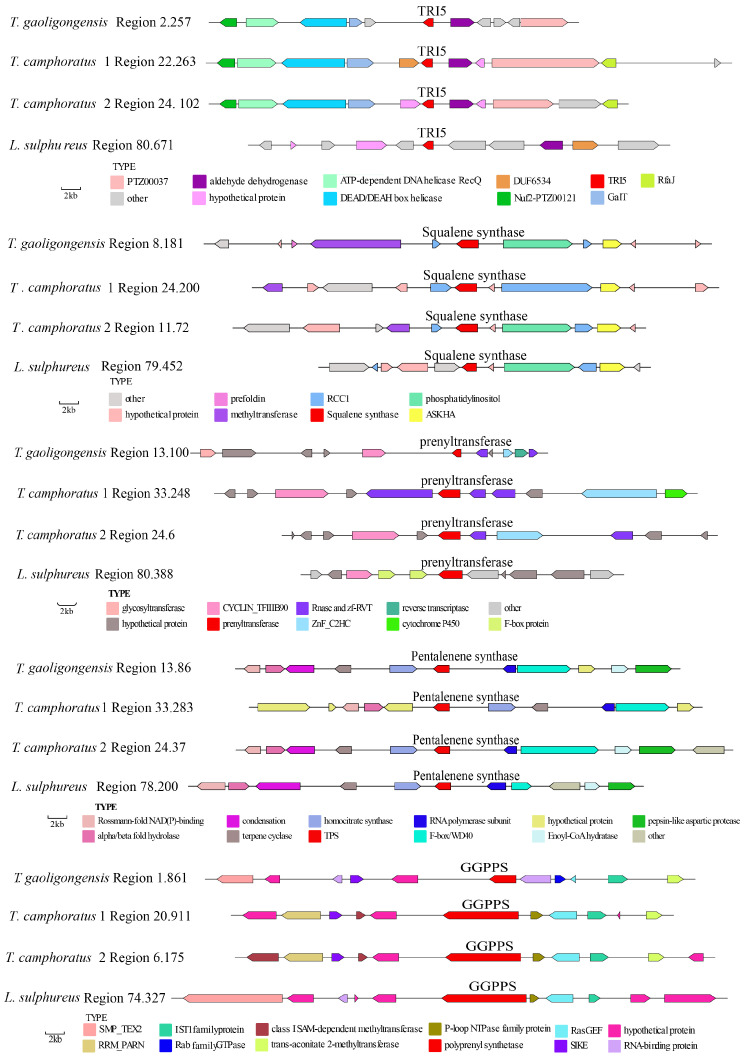
Comparative Analysis of Genes Flanking Various Types of TPS in *L. sulphureus* and Fungi of the *Taiwanofungus* Genus.

*L. sulphureus* exhibits similarities to *Taiwanofungus* fungi in the number and types of TPS and contains four PKS domains with identical structures. To investigate the phylogenetic relationship between *L. sulphureus* and fungi of the *Taiwanofungus* genus, a comparative analysis was conducted on the highly homologous TPS types from the genomes of the four species. The *TRI5* gene clusters in fungi of the *Taiwanofungus* genus (*T. gaoligongensis* Region2.257, *T. camphoratus1* Region22.263, *T. camphoratus2* Region24.102) exhibit high similarity, with each containing Nuf2-PTZ00121, ATP-dependent DNA helicase RecQ, DEAD/DEAH box helicase, GalT, and aldehyde dehydrogenase. In contrast, the TRI5 gene (Region80.671) of *L. sulphureus* shares only aldehyde dehydrogenase with the fungi of the *Taiwanofungus* genus. The *PentS* gene clusters (*T. gaoligongensis* Region13.86, *T. camphoratus1* Region33.283, *T. camphoratus2* Region24.37, *L. sulphureus* Region78.200) in all 4 strains contain alpha/beta fold hydrolase, homocitrate synthase, Rossmann-fold NAD(P)-binding proteins, terpene cyclase, RNA polymerase subunits, and F-box/WD40. However, the Ptase modification genes vary significantly among the four strains, with only one shared CYCLN-TFIIIB90 gene. The *Ptase* gene clusters in fungi of the *Taiwanofungus* genus include three Rnase and zf-RVT genes. The *GGPPS* gene clusters in all four strains contain IST1 family proteins and RasGEP genes. Furthermore, the *SQS* modification genes in all 4 strains possess two RCC1 and one ASKHA, with fungi of the *Taiwanofungus* genus additionally containing a common methyltransferase. Although the modification genes for the five TPS types are similar among the four strains, notable differences are evident. For instance, *TgTRI5* and *TgPtase* are significantly smaller than those in the other strains. Moreover, gene inversions and horizontal gene transfers are observed within gene clusters, leading to variations in the size, position, and orientation of even identical modification genes (Figure 12). The similarities and differences in gene clusters between *L. sulphureus* and fungi of the *Taiwanofungus* genus reflect their evolutionary conservation and diversity at the genetic level, suggesting a shared evolutionary origin in secondary metabolic pathways while highlighting distinct evolutionary paths and adaptation mechanisms.

### 3.7. Identification and Analysis of Transcription Factors in *T. gaoligongensis*

**Figure 13:**
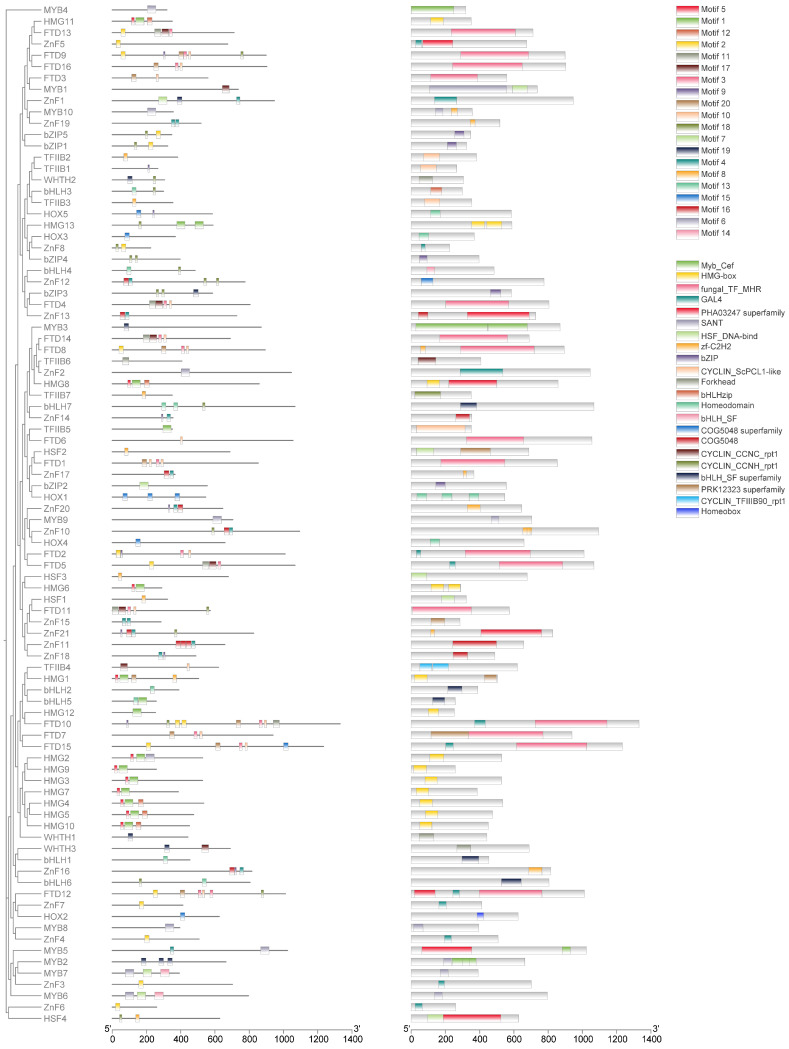
Structural Characterization of the 10 TF Families in *T. gaoligongensis*. *From left to right:* Phylogenetic Tree of Proteins, Conserved Motif Analysis and Conserved Domain Analysis.

A comparative analysis identified a total of 91 TF sequences in the *T. gaoligongensis* genome, categorized as follows: bHLH (7), HSF (4), HOX (5), TFIIB (7), HMG (13), MYB (10), bZIP (4), WHTH (3), FTD (16), and ZnF (21). These sequences were designated as TgbHLH1 through TgZnF21. A phylogenetic tree was constructed for these 10 families of TFs using MEGAX64, revealing 15 subgroups. Members within the same subgroup exhibited high similarity in their conserved motif compositions, suggesting shared functional characteristics. MEME motif analysis identified 20 motifs, and members of the same family generally contained similar motifs. Phylogenetically related members typically shared similar motifs; notably, 11 TgHMG proteins all contained motif5, suggesting that motif5 may represent a characteristic conserved motif of the TgHMG TF family. Furthermore, TFs from different families exhibited variations in motif types, numbers, and distributions, consistent with the results of the phylogenetic analysis. These differences are likely related to specific functions within each family. Analysis of conserved protein structures revealed that all TFs contain typical conserved domains for their respective types, exhibiting high similarity in domain structures within the same subgroup (Figure 13).

### 3.8. Gene Expression Analysis of TgPKS, TgTPS, and TgTFs Under Different Cultivation Conditions

**Figure 14:**
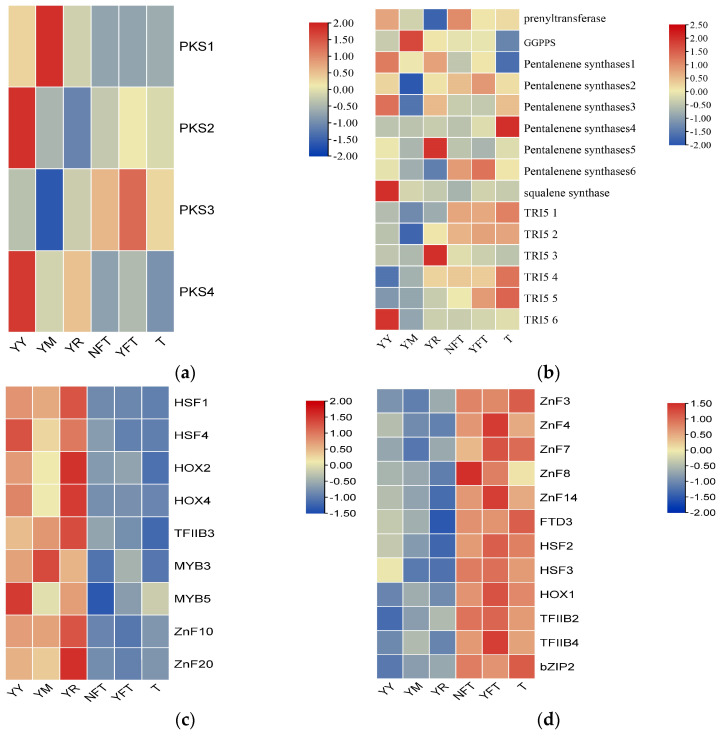
Interactive Heatmap of Gene Expression for (**a**) *TgPKS*, (**b**) *TgTPS*, and (**c**,**d**) *TgTFs* Under Different Cultivation Conditions. T: pea powder (5 g/L), KH₂PO₄ (1 g/L), MgSO₄ (0.5 g/L), yeast powder (5 g/L), and vita-min B1 (0.1 g/L).,NFT: T+Triton X-100 (100 μL) + *C. kanehirae* sawdust (5 g/L), YFT: T+ Triton X-100 (100 μL) + *C. burmannii* sawdust (5 g/L), YY: 15 mL MM medium +4 g Populus alba sawdust, YM: 15 mL MM medium +4 g Zea mays flour, YR: 15 mL MM medium +4 g Coix Coicis Semenurr.

Transcriptome sequencing was conducted on the mycelium of *T. gaoligongensis* under various cultivation conditions. FPKM values were obtained based on gene IDs, and expression data were collected. An interactive heatmap was generated using TBtools software version 2.056 to visualize gene expression differences for PKS, TPS, and TFs across different cultivation conditions. The analysis revealed that, under solid cultivation conditions (YY, YM, YR), the expression levels of *TgPKS1* and *TgPKS4* were significantly higher than those under liquid cultivation conditions (T, NFT, YFT). Conversely, under liquid cultivation conditions, *TgPKS3*, *TgTRI5-1*, *TgTRI5-2*, and *TgTRI5-5* exhibited increased expression, potentially associated with specific biological processes or metabolic pathways in the liquid environment. Additionally, differences in TF expression across various conditions were observed. *TgHOX2*, *TgHOX4*, *TgHSF1*, *TgHSF4*, *TgMYB3*, *TgMYB5*, *TgTFIIB3*, *TgZnF10*, and *TgZnF20* were upregulated under solid cultivation conditions, while *TgbZIP2*, *TgFTD3*, *TgHOX1*, *TgHSF2*, *TgHSF3*, *TgTFIIB2*, *TgTFIIB4*, *TgZnF3*, *TgZnF4*, *TgZnF7*, *TgZnF8*, and *TgZnF14* exhibited more pronounced expression under liquid cultivation conditions (Figure 14).

### 3.9. Quantitative Real-Time PCR Analysis

**Figure 15:**
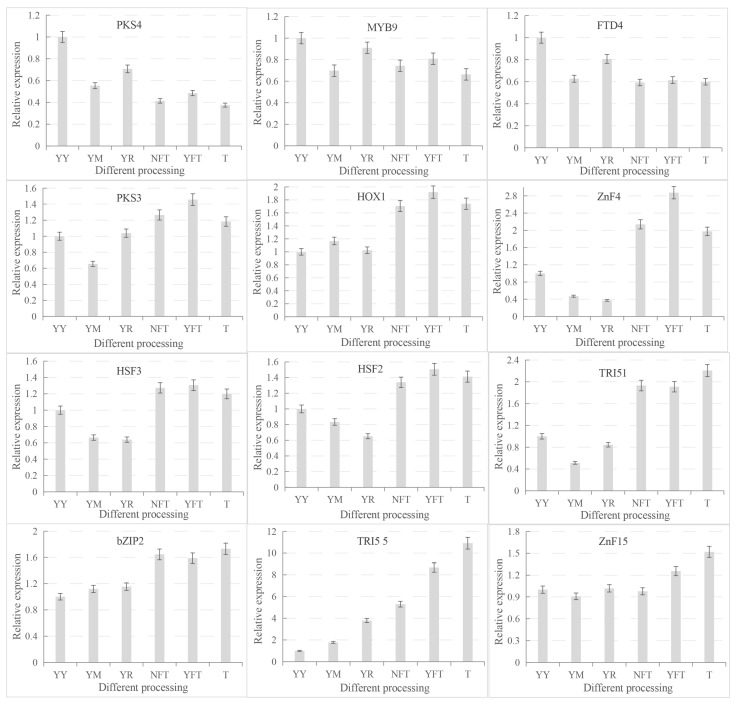
Relative Expression of Differentially Expressed Genes by qRT–PCR. T: pea powder (5 g/L), KH₂PO₄ (1 g/L), MgSO₄ (0.5 g/L), yeast powder (5 g/L), and vita-min B1 (0.1 g/L). NFT: T+Triton X-100 (100 μL) + *C. kanehirae* sawdust (5 g/L), YFT: T+ Triton X-100 (100 μL) + *C. burmannii* sawdust (5 g/L), YY: 15 mL MM medium +4 g Populus alba sawdust, YM: 15 mL MM medium +4 g Zea mays flour, YR: 15 mL MM medium +4 g Coix Coicis Semenurr.

qRT–PCR was utilized to analyze the expression of 12 differential genes related to secondary metabolism in *T. gaoligongensis*, validating the accuracy of the transcriptome sequencing results. The results indicated that the relative expression of these 12 genes was consistent with the trends observed in the transcriptome data. Under solid cultivation conditions (YY, YM, YR), the expression levels of *TgPKS4*, *TgMYB9*, and *TgFTD4* were upregulated, while under liquid cultivation conditions (T, NFT, YFT), *TgPKS3*, *TgHOX1*, *TgHSF2*, *TgHSF3*, *TgZnF4*, *TgTRI5-1*, *TgbZIP2*, *TgTRI5-5*, and *TgZnF15* exhibited increased expression (Figure 15). These findings suggest that cultivation conditions significantly influence the expression patterns of genes associated with SM synthesis in *T. gaoligongensis*. Different cultivation conditions may promote the activation of specific metabolic pathways, thereby enhancing the synthesis of various metabolites.

### 3.10. Prediction of TgTFs Binding Sites in the Promoter Regions of TgPKS and TgTPS Genes

**Figure 16:**
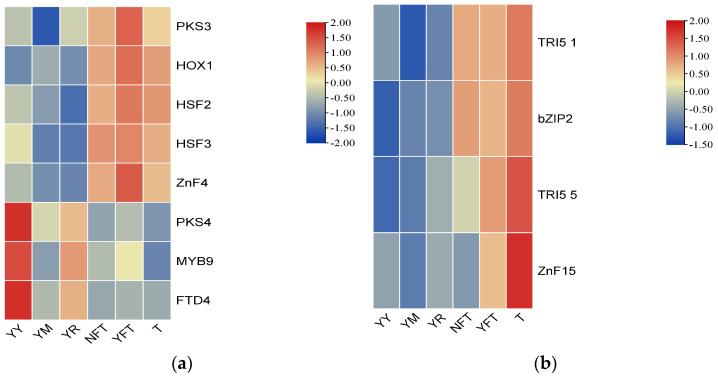
Interactive Heatmap of Gene Expression for (**a**) *TgPKS*, (**b**) *TgTPS*, and Co-expressed *TgTFs* Under Varying Cultivation Conditions. T: pea powder (5 g/L), KH₂PO₄ (1 g/L), MgSO₄ (0.5 g/L), yeast powder (5 g/L), and vitamin B1 (0.1 g/L). NFT: T+Triton X-100 (100 μL) + *C. kanehirae* sawdust (5 g/L), YFT: T+ Triton X-100 (100 μL) + *C. burmannii* sawdust (5 g/L), YY: 15 mL MM medium +4 g Populus alba sawdust, YM: 15 mL MM medium +4 g Zea mays flour, YR: 15 mL MM medium +4 g Coix Coicis Semenurr.

A comprehensive analysis of expression patterns for *TgPKS*, *TgTPS*, and *TgTFs* under different cultivation conditions revealed the following: under solid cultivation conditions (YY, YM, YR), *TgMYB9* and *TgFTD4* exhibited high expression levels closely correlated with *TgPKS4*. Under liquid cultivation conditions (T, NFT, YFT), *TgHOX1*, *TgHSF2*, *TgHSF3*, and *TgZnF4* displayed similar expression patterns to *TgPKS3*, all exhibiting an upregulation trend. Additionally, the TPS *TgTRI5-1* and TF *TgbZIP2* exhibited a similar high-expression pattern under liquid cultivation, while the *TgTRI5-5* and *TgZnF15* exhibited similar high expression under the same conditions (Figure 16). Based on these findings, it is hypothesized that these *TgTFs* may regulate the *TgPKS* and *TgTPS* genes. To test this hypothesis, TBtools software version 2.056 was used to identify the promoter regions (2000 bp) of the *TgPKS* and *TgTPS* genes based on the complete genome data of *T. gaoligongensis*.

A detailed analysis of TF binding sites in the promoter regions of *TgPKS* and *TgTPS* genes was conducted using the JASPAR database. Several potential binding sites with high relative scores were identified, indicating their involvement in the regulation of *TgPKS* and *TgTPS* genes. *TgHSF2* binds to the promoter region of *TgPKS3* at positions 843 to 850 on the positive strand, with the binding site “atggaata” showing a high relative score of 0.956669333, indicating a role in regulating *TgPKS3. TgHSF3*, *TgHOX1*, and *TgZnF4* also demonstrate a strong match with the promoter region of *TgPKS3*. The binding site for *TgMYB9* is “ttgtcatcgc”, and for *TgFTD4* it is “cgcgcaatagccttc”, both of which exhibit a strong match with the promoter region of *TgPKS4*. *TgbZIP2* binds to the promoter region of *TgTRI5-1* at the site “aagcat”, demonstrating high specificity. The binding site for *TgZnF15* with *TgTRI5-5* is “tgccaag” (Table 4). These findings indicate that these *TgTFs* may bind to DNA sequences in the promoter regions of the corresponding *TgPKS* and *TgTPS* genes, potentially activating their expression.

## 4. Discussion

*T. camphoratus* is known for its abundant bioactive compounds, including the polyketide Antrocamphin A and the terpenoids Antcin A and Antcin C. These components exhibit a range of clinical effects, including anticancer, antioxidant, anti-inflammatory, immunomodulatory, and antimicrobial activities. Rapid advancements in modern sequencing technologies have significantly accelerated research on the genomes of *T. camphoratus* and other fungi. The first genome of *T. camphoratus* was sequenced using NGS technology [16]. Chen et al. sequenced *T. camphoratus* using PacBio SMRT and Illumina MiSeq paired-end methods, revealing its complex reproductive system and genetic features; however, SMs were not analyzed [17]. This study integrates sequencing data from ONT and Illumina NovaSeq to successfully assemble the complete genome of *T. gaoligongensis*. This research elucidates the fundamental genomic features of *T. gaoligongensis* and 11 other fungal species, comparing them in terms of genome size, scaffold N50, contig N50, and GC content.

**Figure 17:**
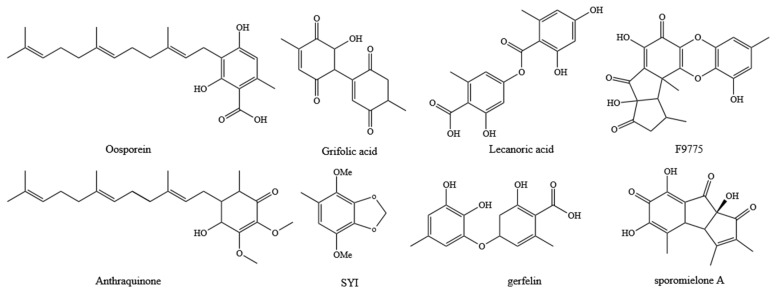
Derivatives of orsellinic acid in fungi.

Fungal SMs are widely utilized in biomedicine, biocontrol, and the food industry, with most biosynthesis closely linked to the catalytic activities of PKS or TPS enzymes [1]. In this study, we performed genomic mining of *T. gaoligongensis* and 11 other fungal species, identifying putative SM biosynthesis gene clusters. AntiSMASH analysis identified 25 putative SM gene clusters in the *T. gaoligongensis* genome, including 4 PKS gene clusters, 6 NRPS gene clusters, and 15 TPS gene clusters. In *Aspergillus nidulans*, the gene cluster F9775 responsible for orsellinic acid synthesis contains an NR-PKS with the domains SAT-KS-AT-PT-ACP-ACP-TE [44]. In this study, *TgPKS1*, *T. camphoratus* Region30.386, *T. camphoratus* Region31.16, *L. sulphureus* Region81.432, *F. palustris* Region66.13, and *D. quercina* Region38.26 possess NR-PKSs with identical domain structures. *TgPKS1* shares 100% sequence homology with the PKS gene responsible for orsellinic acid synthesis in *T. camphoratus*, suggesting its involvement in the synthesis of orsellinic acid or its derivatives. However, the modification genes of *TgPKS1* differ from those of F9775, indicating significant variations in the orsellinic acid BGCs among different species. PKSs involved in orsellinic acid synthesis are widely distributed across various fungi, producing a range of derivatives, including lecanoric acid in lichens [45], oosporein in *Beauveria bassiana* [46], F9775 and gerfelin in *Aspergillus nidulans* [44], griseofulvin in *Penicillium* species [47], and sporormielone in *Sporormiella* species [48]. Derivatives of orsellinic acid in *T. camphoratus* include anthraquinone and benzodioxol compounds, including SY1 [49] (Figure 17). Currently reported PR-PKSs fall into two categories: one associated with 6MSA, with the domain organization KS-AT-DH-KR-ACP(-TE) [50], and the other associated with hispidin synthesis, with domain organizations CaiC-ACP-KS-AT-DH-KR-ACP-ACP or CaiC-ACP-KS-AT-ACP [51]. In this study, BGCs with the domain organization KS-AT-DH-KR-ACP-TE were identified in *T. gaoligongensis* and nine other fungal species, showing 96% homology with known PKS genes involved in 6MSA synthesis, suggesting that TgPKS2 is associated with the synthesis of 6MSA or its derivatives. The PKS modification genes in *Taiwanofungus* species are identical to those involved in 6MSA synthesis, indicating that PKSs in this genus participate in conserved biosynthetic pathways and regulatory mechanisms. However, the PKSs in seven other BGCs exhibit significant variation in modification genes, reflecting differences in the biosynthetic systems of fungi from different genera in response to environmental adaptation or specific metabolic needs. The domain organization of *TgPKS3*, *T. camphoratus* Region31.107, *T. camphoratus* Region6.374, *D. quercina* Region95.22, *L. sulphureus* Region76.452, and *W. cocos* Region65.71 includes KS-AT-KR-ACP-TE. Structural analysis suggests that *TgPKS3* is evolutionarily related to 6MSA but lacks the DH domain, possibly representing a novel PR-PKS. Additionally, a PKS with the domain organization KS-AT-DH-MT-ER-KR-ACP-TE was identified in the genomes of Taiwanofungus species and *L. sulphureus*. This PKS contains the KR and ER domains characteristic of HR-PKSs, as well as a TE domain, indicating that the *TgPKS4* gene encodes a novel HR-PKS.

Terpenoids are the primary metabolites of *T. camphoratus*, including the sesquiterpene antrocin, triterpenoid antcins, and meroterpenoid antroquinolols [52]. Following the publication of the *T. camphoratus* genome, several class I TPS, a Ptase, and two UbiA-type TPS capable of synthesizing (+)-(S, Z)-α-bisabolene have been characterized through heterologous expression [16,53,54]. However, a comprehensive understanding of other enzymes involved in terpenoid biosynthesis in *T. camphoratus* remains elusive. In this study, we identified six *TRI5* genes in the *T. gaoligongensis* genome. These genes contain several conserved regions: the DDXXX motif, crucial for Mg2+ chelation and substrate binding, which is common in sesquiterpene, diterpene, and monoterpene synthases; the NDXXSFYKEEL region, associated with pyrophosphate interaction, where the residues N, S, and E are critical for catalytic activity [55]; and the XXRYRL motif, involved in the interaction between farnesyl and pyrophosphate, present in sesquiterpene and bisabolene synthases from *Fusarium* species [56]. Clustering analysis suggests that *TgTRI5* likely produces tetracyclic sesquiterpene type B tricothecene compounds [57]. Sesquiterpenes are synthesized by sesquiterpene synthases that convert farnesyl pyrophosphate into a sesquiterpene scaffold, which is subsequently modified through various reactions [58]. The amino acid sequences of sesquiterpene synthases are highly conserved, with motifs such as DXXXD or DDXXD playing a crucial role in metal ion binding [59]. All six *TgPentS* enzymes identified contain the conserved DXXXD sequence, suggesting they may catalyze the production of δ-cadinol [60], β-copaene [61], and α-muurolene [62] or their derivatives. SQS is essential for triterpene biosynthesis, catalyzing the condensation of two FPP molecules to produce squalene [63]. Phylogenetic analysis indicates that *TgSQS* is closely related to *SQS* in the *Polyporaceae* family but more distantly related to *SQSs* from non-*Polyporaceae* fungi, suggesting a trend toward centralized evolution of *SQS* genes in fungi [64]. Ptases are responsible for the prenylation of isoprenoid compounds. They are classified into TPSs and protein Ptases [65]. Our study identifies *TgPtase* as a protein Ptase that covalently attaches isoprenoid groups to cysteine residues, a modification known as prenylation [66]. Additionally, we identified *TgGGPPS* in the *T. gaoligongensis* genome, which catalyzes the conversion of IPP to GGPP, providing essential precursors for the synthesis of carotenoids, diterpenes, chlorophyll, and ubiquinones [67].

*T*. *gaoligongensis* Z. H. Chen and Y. M. Yang is named for its morphological similarity to *T. camphoratus* Sheng H. Wu, Z. H. Yu, Y. C. Dai and C. H. Su. *T. gaoligongensis* shares similar growth characteristics and morphology with *T. camphoratus*. The fungal rDNA sequence was uploaded to NCBI, showing close relatedness to *T. camphoratus*. Under identical culture conditions, both fungi exhibit smooth, hoof-like shapes; they appear bright red initially, gradually turning pale reddish-brown with growth [22]. The relative lengths and arrangements of gene fragments influence species evolution and growth. In *T. gaoligongensis*, synteny with close relatives reveals differences in gene fragment lengths and positions, indicating gene rearrangements. These differences may contribute to species divergence and lead to distinct evolutionary trajectories. Compared to the genome of *T. camphoratus*, the genome of *T. gaoligongensis* exhibits some gene fragment deletions, despite no changes in gene order. These deletions may result in differences in protein-coding functions between the two genomes, influencing their evolutionary trajectories. Research on the mitochondrial genome of *T. gaoligongensis* revealed that, although it forms an independent branch closely related to *Taiwanofungus* species, it exhibits a 4209 bp fragment deletion consistent with the findings of this study [22]. Furthermore, *L. sulphureus* and *Taiwanofungus* species both belong to the Polyporaceae family, exhibiting high similarity in SM biosynthesis gene clusters. Both *L. sulphureus* and *T. camphoratus* possess a four-level mating system [17,68]. These findings indicate a phylogenetic relationship between *T. gaoligongensis* and *L. sulphureus*, suggesting they share similar secondary metabolic pathways and ecological functions.

SMBGCs in fungi can be activated by adjusting cultivation conditions or through genetic modifications. Different media and cultivation methods significantly influence the growth rate and SMs of *T. camphoratus* [69]. Gene expression under varying cultivation conditions can result in the production of different compounds. Chen et al. demonstrated that different wood substrates significantly affect the expression levels of genes related to terpene biosynthesis in *T. camphoratus* mycelium. For instance, on a *Cinnamomum kanehirae* substrate, the expression levels of 2,3-oxidosqualene cyclase (OCS), SQS, and squalene epoxidase (SE) were significantly increased [70]. In this study, the expression of *TgPKS1*, *TgPKS4*, and nine *TgTFs* was notably upregulated under solid cultivation conditions (YY, YM, YR), while *TgPKS3*, *TgTRI5-1*, *TgTRI5-2*, and twelve *TgTFs* showed significant expression under liquid cultivation conditions (T, NFT, YFT).

TFs play a crucial role in the regulatory network of secondary metabolism in fungi through synergistic regulation [71]. For instance, in *Aspergillus nidulans*, inducing the expression of *apdR* successfully activated a PKS/NRPS hybrid biosynthetic gene cluster, leading to the production of Aspyridones with moderate cytotoxic activity [72]. AflR, a transcriptional activator for aflatoxin, recognizes sequences in the promoters of most sterigmatocystin and aflatoxin gene clusters and positively regulates these clusters [73,74]. These results are consistent with our study findings. In our research, we integrated co-expression patterns under different cultivation conditions with TF binding site predictions and hypothesized that *TgFTD4* and *TgMYB9* may directly regulate the expression of *TgPKS4* by binding to its promoter region. The expression of the *TgPKS3* is likely regulated by *TgHOX1*, *TgHSF2*, *TgHSF3*, and *TgZnF4*. Additionally, *Tg bZIP2* may positively regulate the expression of *TgTRI5-1*, while *TgZnF15* may positively regulate the expression of *TgTRI5-5*. This regulation could enhance the transcription of TPS genes, promoting the synthesis of TPSs and the subsequent production of sesquiterpene compounds.

## 5. Conclusions

This study utilized Illumina NovaSeq and ONT for the whole-genome sequencing and annotation of *T. gaoligongensis*, and performed genomic comparisons with 11 other fungal species. The research elucidated the complete genomic sequence of *T. gaoligongensis* and predicted a wealth of functional genes. Comparative genomics revealed differences in genome size and GC content between *T. gaoligongensis* and other fungal species. AntiSMASH analysis identified a variety of SM gene clusters in these species. Genes such as *TgPKS1*, *TgPKS3*, *TgTRI5*, *TgPentS*, and *TgSQS* exhibited high similarity to previously characterized gene clusters. Transcriptomic analysis assessed the effects of different cultivation conditions on the expression of *TgPKS*, *TgTPS*, and *TgTF* genes, revealing that these conditions can induce the expression of specific genes. Co-expression patterns and TF binding site predictions indicated that *TgTFs* may positively regulate the expression of *TgTPS* and *TgPKS* genes. These findings provide novel strategies for genomic exploration, including gene silencing, heterologous expression, promoter regulation, and mutation induction, to activate silent BGC biosynthesis, develop new bioactive SMs, and advance drug research and development.

## Figures and Tables

**Table 1:** Genomic Characteristics of the 12 Strains.

Strain	Accession Number	Total Length (Mb)	Scaffold	GC Content (%)	PKS	NRPS	TPS
*Taiwanofungus gaoligongensis*	GCA_037127245.1	34.58	19	50.72	4	6	15
*Taiwanofungus camphoratus1* (M8)	GCA_003999685.1	33	14	51	4	6	15
*Taiwanofungus camphoratus2* (monokaryon S27)	GCA_000766995.1	32.2	360	50.5	4	4	15
*Daedalea quercina* (L-15889)	GCA_001632345.1	32.7	357	55	5	2	13
*Wolfiporia cocos* (MD-104)	GCA_000344635.1	50.5	348	52	5	3	12
*Laetiporus sulphureus* (gfLaeSulp1.1)	GCA_927399515.1	37.4	14	51.5	5	5	16
*Fibroporia radiculosa* (TFFH 294)	GCA_000313525.1	28.4	861	51	6	1	11
*Fomitopsis palustris* (ATCC 62978)	GCA_001937815.1	43.9	226	55.5	5	2	5
*Fomitopsis schrenkii* (FP-58527 SS1)	GCA_000344655.2	41.6	504	55.5	6	1	8
*Fomitopsis betulina* (CIRM-BRFM 1772)	GCA_022606075.1	42.9	251	53.5	4	4	8
*Postia placenta* (MAD-698-R-SB12)	GCA_002117355.1	42.5	549	54	5	4	17
*Neoantrodia serialis* (Sig1Antser10)	GCA_022376445.1	66.7	893	56	9	7	13

**Table 2:** Genome Assembly of *T. gaoligongensis*.

Item	Value	Item	Value
Total length (Mb)	34.58	Scaffolds	19
Max length (bp)	4247058	Contigs N20	3,457,967
GC content (%)	50.72	Scaffolds N20	3,457,967
Gene number	7955	Contigs N50	2,343,139
Total gene number (bp)	14786310	Scaffolds N50	2,343,139
Gene/Genome (%)	42.61	Contigs N90	1,499,237
Contigs	19	Scaffolds N90	1,499,237

**Table 3:** Functional Annotation of the *T. gaoligongensis* Genome.

Item	NR	SwissProt	KEGG	GO	eggNOG	P450	TCDB	Pfam
Count	7548	5204	2874	5148	6700	7768	944	5613
Percentage (%)	94.88	65.41	36.13	64.71	84.22	97.65	11.87	70.56

**Table 4:** Binding Sites of Co-expressed *TgTFs* in the Promoter Regions of *TgPKS* and *TgTPS*.

TPS ID	Score	Relative Score	Squence ID	Start	End	Strand	Predicted Sequence
TgHSF2	11.00965	0.956669333	TgPKS3	843	850	+	atggaata
TgHSF3	8.580054	0.999999993	TgPKS3	239	244	+	ggccat
TgHOX1	9.405297	0.949936448	TgPKS3	1925	1931	+	cgaaaca
TgZnF4	10.068156	0.889975916	TgPKS3	1677	1691	+	cggacaagtgcctgc
TgMYB9	11.1348505	0.915335488	TgPKS4	124	133	+	ttgtcatcgc
TgFTD4	9.56837	0.882805555	TgPKS4	1351	1365	-	cgcgcaatagccttc
TgbZIP2	9.614646	1.00000001	TgTRI5-1	631	636	+	aagcat
TgZnF15	11.982402	0.99611025	TgTRI5-5	1604	1610	+	tgccaag

## Data Availability

This study project is available under NCBI BioProject accession number PRJNA1051983 and BioSample accession number SAMN38809687. The complete genome assembly of *T. gaoligongensis* strain YAF008 is available under GenBank accession number JAZIAZ000000000. https://www.ncbi.nlm.nih.gov/datasets/genome/GCA_037127245.1/.

## Supplementary Materials

The following supporting information can be downloaded at: https://www.mdpi.com/article/10.3390/jof10120826/s1. Figure S1. Phylogenetic relationship between PKS1 of 6 genomes and putative orsellinic acid biosynthesis core genes; Figure S2. Phylogenetic relationship between PKS2 of 10 genomes and putative 6MSA biosynthesis core genes; Figure S3. The protein alignment of TgTRI5 from *T. gaoligongensis* and other related fungal TRI5 proteins; Figure S4. The protein alignment of TgPentS from *T. gaoligongensis* and other related fungal PentS proteins; Figure S5. The protein alignment of TgGGPPS from *T. gaoligongensis* and other related fungal GGPPS proteins; Figure S6. The protein alignment of TgPTase from *T. gaoligongensis* and other related fungal PTase proteins; Figure S7. The protein alignment of TgSQS from *T. gaoligongensis* and other related fungal SQS proteins; Table S1. Primer list of genes for qRT-PCR.
