# Supplementary material for: Genomic Features of Taiwanofungus gaoligongensis and the Transcriptional Regulation of Secondary Metabolite Biosynthesis
Source: J Fungi (Basel). 2024 Nov 27;10(12):826. doi: 10.3390/jof10120826 (PMC11678574; doi:10.3390/jof10120826)
Supplement: Supplementary file 1 [file jof-10-00826-s001.zip › jof-3297590-supplementary.pdf]

Supporting Information

Table S1. Primer list of genes for qRT-PCR.

Figure S1. Phylogenetic relationship between PKS1 of 6 genomes and putative orsellinic acid biosynthesis core genes.

Figure S2. Phylogenetic relationship between PKS2 of 10 genomes and putative 6MSA biosynthesis core genes.

Figure S3. The protein alignment of TgTRI5 from *T. gaoligongensis* and other related fungal TRI5 proteins.

Figure S4. The protein alignment of TgPentS from *T. gaoligongensis* and other related fungal PentS proteins.

Figure S5. The protein alignment of TgGGPPS from *T. gaoligongensis* and other related fungal GGPPS proteins.

Figure S6. The protein alignment of TgPTase from *T. gaoligongensis* and other related fungal PTase proteins.

Figure S7. The protein alignment of TgSQS from *T. gaoligongensis* and other related fungal SQS proteins.

Table S1.

Primer Name	Sequence（5'-3'）
TgZnF4 F	TTACAGCGAACAGGCCAATG
TgZnF4 R	TTCGTGCAGTACGCCATGAT
TgPKS4 F	ATAGGTGGAATGCTGAAGCA
TgPKS4 R	GATGCATCTGACTAGCCTGA
TgbZIP2 F	AGCTTGGACAGTACGTCATA
TgbZIP2 R	CGTTGAGCAGCACGATTCTG
TgZnF15 F	CTAGACCTAGCACGCTACGG
TgZnF15 R	TCGTCGACCGCATGTATCAC
TgHOX1 F	GCTCATTGTGCGTAACAGGG
TgHOX1 R	AGATGCGCAGGTGCTCGTCG
TgFTD4 F	ATGCAGAGCGGCAAAGGTTC
TgFTD4 R	TCGAATATACATCTGCAGGA
TgHSF2 F	GATACGCTGGTCCGAGAGTG
TgHSF2 R	TGTGGAATCCGTACATGTTC
TgHSF3 F	TCGGATGATAACCAGTTTGG
TgHSF3 R	CAGCACGTTCTGGTAGTTGT
TgTRI5-1 F	GAATGTAGCAGACCAGTACC
TgTRI5-1 R	TCGACAGTAGGAAGATGGCT
TgMYB9 F	ACGATTCCGATATGGATATG
TgMYB9 R	AGATGTTGATTCATCTGCCA
TgPKS3 F	GTCATGTCCTTGAGCATTCG
TgPKS3 R	TATGAGACCCGATTCGCGAT
TgTRI5-5 F	TTCTAGGCTCGATCGACTCG
TgTRI5-5 R	GCTACCAGACTCGTTCTCGA

Figure S1.

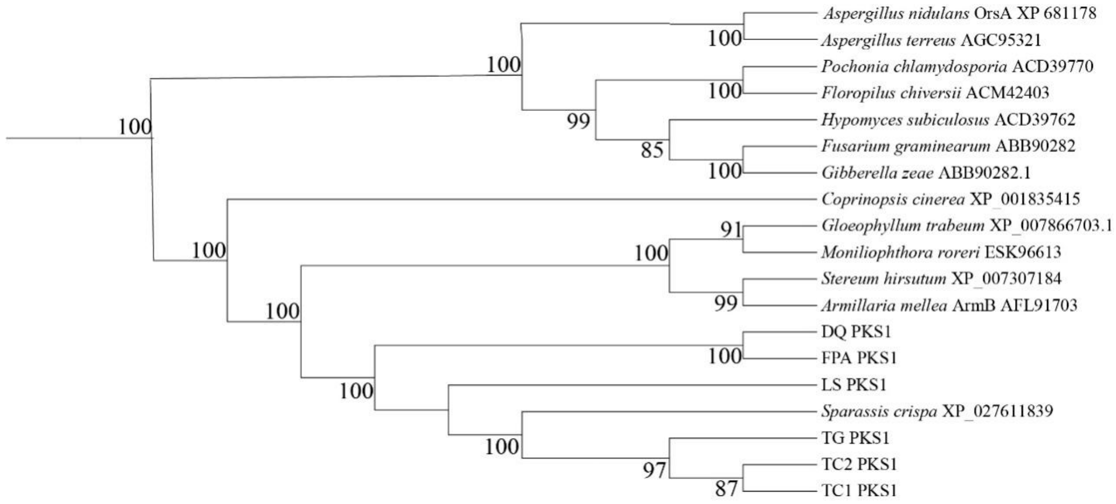

Figure S2.

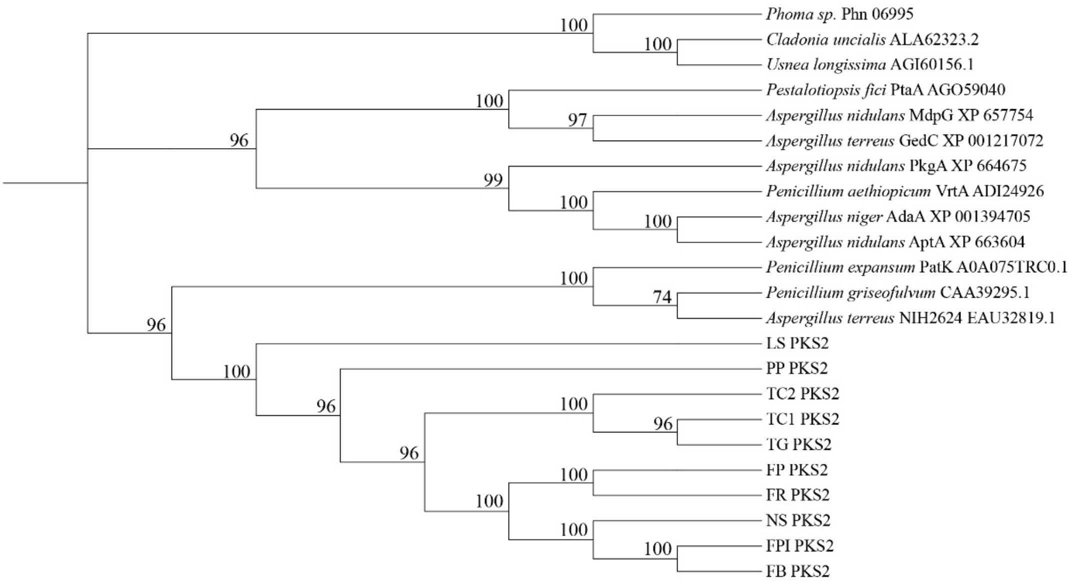

Figure S3.

```
TRI5_2                              GQEGLAAVSKQAICEFLDRLNFT.FPEYKRDLELEARVNE     66
TRI5_6                              HQETIASVSQQTIRDFLDKISLT.FPDYKHDLELETRVKE     79
TRI5_5                              NINVTKAVSREAIHGFFDKIDFK.CPGYKRDAELELQVKE     54
TRI5_4                              ........................................      0
TRI5_3                              DQKSIAAISQRAVRDLLDRLNLS.ISTYERDYELERRVKE     41
TRI5_1                              ENQTSTRDIKEAICDFIDKTNLSALPEYKRDLELESRIKD     67
Sparassis_crispa_XP_027613108.1     NEASLLAVSQQVIRDFLDRMNVT.IPPYLPDLEVMSRVYK     66
Beauveria_bassiana_XP_008596951.    DTNISKAAFAAKAADFIQRMGFDMRASNPRDFELEDTVLA     53
Trichoderma_reesei_XP_006964535.    EYVANHVRNLVAMMGFDMRKSLR.....DRDLELEAKVMD     38
Trametes_versicolor_XP_008037460.   ASVDAIAEIKDTLKDFLRRLNYR.PPHTPANAKLRAEVTA     54
Consensus

TRI5_2                              ATRGRENGE.....YMRPYVVTAILITVMAYGHLSSIDAK    101
TRI5_6                              IVHSWGYGE.....DIRPHVVTALILTTTAYSHIASMDTK    114
TRI5_5                              VVQSWRNES.....LIRSHVVTALIGTITAYGHISSMDTK     89
TRI5_4                              ........................................      0
TRI5_3                              TIRSWDAET.....LLSPYLSPAIILTITAFSHIRNFETR     76
TRI5_1                              VTKFRANEE.....TMRPYITVGVAMAVTAYSHLPTVEAK    102
Sparassis_crispa_XP_027613108.1     VLQTWDFGD.....AIRPHVITALTITITAYNHLTDIEAK    101
Beauveria_bassiana_XP_008596951.    KVRDLGIQDSQLEPLLRVHEQACLFTTLCNRSACQ..NLK     91
Trichoderma_reesei_XP_006964535.    KLMELGLSSTDSKKPLERFLKSGCLLASLYYRHHP.TDAK     77
Trametes_versicolor_XP_008037460.   EIVSWNADLSPS..FMHGLAETCYTIAESAYAHTS.YEHQ     91
Consensus

TRI5_2                              LAVALYTALLVALDDPAVFDSLSAQKLPFRLCAGSAQRDG    141
TRI5_6                              VQITLFTTLIIAFDDPAILNALASSEFHQRVCTGAAQRGE    154
TRI5_5                              VQIALFTVLVLAMDDPAILGSIDSHQFHQRVCAGEAHRDT    129
TRI5_4                              ........................................      0
TRI5_3                              VHIALFGVLVIAMDDPAVLDSVASRDFHRKLCTGALQSDP    116
TRI5_1                              LQIVLYTAILAYLDDPAVFDSLGAGNFHRRLCAGSVQHDT    142
Sparassis_crispa_XP_027613108.1     VQIALFTAITTALDVPVIMNSVSWRDLHTLFLPCSAENRS    141
Beauveria_bassiana_XP_008596951.    VSLAVFTELIWMVEEHVV.DKSSAKQFTFNLMNNYPQTDP    130
Trichoderma_reesei_XP_006964535.    VYTAIHAILLLGVDDYFS.DVSLTRTFAHKFGTRKAQGHP    116
Trametes_versicolor_XP_008037460.   RIIALYTTYLTYVDDLGGRDLDALGEFGRRLLAREALGDP    131
Consensus

TRI5_2                              DVLGELSRNLAGMWGIYPDFAASAVCTSALRFVNACFLEN    181
TRI5_6                              DMLGKFMRVLSGMWEHYAPFAANSIFASALRFVNVSILEN    194
TRI5_5                              GMLGEFTRIISGMWTHYPPFSANLIFASALCFVNMSMLEN    169
TRI5_4                              ............MWDHFPKSAANPIFVSTLRWINGTVLEN     28
TRI5_3                              NILGEFTRNLAGMWDHFPPFSASSIFTSALQFVNGCILER    156
TRI5_1                              NILGALATNMVEMFACFPRFSASSILTSTLRFVNGSMIEN    182
Sparassis_crispa_XP_027613108.1     GLVRELRGILVKMWDHYPPCSASAIFSSVLKFISATIFEN    181
Beauveria_bassiana_XP_008596951.    YLEALAKYLKVEVGHDLGDFRTGILLASWIDTLNSMVLES    170
Trichoderma_reesei_XP_006964535.    LLDCVARLLRDETPKIFGPFTTNMIITSTLDGINGFSLEA    156
Trametes_versicolor_XP_008037460.   .ALDRLVTNLQDMYAYYPRLSAHSIAVSTLDFFVGSYVEA    170
Consensus                                                      s          e

TRI5_2                              MGT.DAMLRPGMRSFVDYQRGMSGVADAYACFIWEKAR..    218
TRI5_6                              ESG.DMKLHSEALPFIEYRRSMSATAEAYACFIWDKEK..    231
TRI5_5                              ESG.SVELCSAALPFVQYRRSRSATAEAYACFIWEKAE..    206
TRI5_4                              ESQ.DMILTFSALSFIEYRRSMSGAPEAFAAFIWEKAR..     65
TRI5_3                              AAQKEMQLSSDALPFITYRRNMSGLAEAFGYFIWEKDV..    194
TRI5_1                              TCG.CALLSPHSLSFVKYRRELTGIAEAYAAFIWEKSK..    219
Sparassis_crispa_XP_027613108.1     ETK.AIALSSDGAPFIEYRRAKGGLPEAYAYFIWEKAK..    218
Beauveria_bassiana_XP_008596951.    ELA..ENRATTVLGYHTWIRANTGGGAAVCQFLIPTEQD.    207
Trichoderma_reesei_XP_006964535.    TFP..RGFLRAMPGFSCWLRSCTGWSAAYACFIFPNTKFP    194
Trametes_versicolor_XP_008037460.   TGK.GMAVAPGATKYPAYMRMKTGIGAAYALLNFVKDWR.    208
Consensus                                              r       a

TRI5_2                              .FPDVRIYLEAMLDAMLFIGRSNDVLSFYKEELAGETGNY    257
TRI5_6                              .FPDVQLYMQAIPDVMLYVGYVNDILSFYKEELAGEKVNY    270
TRI5_5                              .FPDVHVYLQAIPDVMLFIGSVNDILSFYKEEIAGETGNY    245
TRI5_4                              .FPDVSVYVQAIPDALLYINYVNDILSFYKEELQNETGNY    104
TRI5_3                              .FPDVEVYMQAIPDIIWYQNHVNDILSFYKEELAGEKGNY    233
TRI5_1                              .FPDVGVYIQALPYVMQYLAYANDVMSFYKEELVGEVDNY    258
Sparassis_crispa_XP_027613108.1     .FPDIKVYVQAIPHAMRYINYVNDILSFYKEELEGETGNY    257
Beauveria_bassiana_XP_008596951.    FEKNCFEYMQLTAAVNDMINYVNDVLSFYKEWIQRKEDCF    247
Trichoderma_reesei_XP_006964535.    ESEYLGRYVQIMPNLRDIICYVNDILSFYKERVVAKEGCF    234
Trametes_versicolor_XP_008037460.   .DPEDLFYLQLIPEIEFYTDTINDILSFYKESLAGETDNL    247
Consensus                                   y              nd  sfyke

TRI5_2                              IHDCALVTGESVLHTLQHLIDDILAALARVRTVLGEG..E    295
TRI5_6                              IHDRALVTEKTVPETLLDVTDETVAAVGRIRTILGEG..E    308
TRI5_5                              IHDRASITGKTASYTLREVVDETVAAVARIRSILGEG..K    283
TRI5_4                              IQDRAAVTGRSALETLREVINDTVVAMGRIRAILGEG..E    142
TRI5_3                              MGDRAITTGKPTLETLRDVIDETIIITERIRKILGEG..P    271
TRI5_1                              VHGRAAATGKPVLEAFRDTVDETVAASDRVRTILGEG..E    296
Sparassis_crispa_XP_027613108.1     IGDRGLVEGKSALEVLREVIDETVGAAERVRRILGEG..E    295
Beauveria_bassiana_XP_008596951.    ITQISRENSCTVMESLDWLLEHTVTLYLNHCSVMATFPGN    287
Trichoderma_reesei_XP_006964535.    ISNLAQEKSMDDYSTLALVCDYVLSLDKEIREFLAEDE.I    273
Trametes_versicolor_XP_008037460.   IHLRAAAEQKDPLSVLREVVEETLESIRKVEVLTAADP.Q    286
Consensus

TRI5_2                              AREAWEDFAAGYVYFHILSSRYRLNDVLEDQCIVDLGLS.    334
TRI5_6                              ARDAWENFATGYISVHTGNPRYRLQEIIGGKYIMDVASY.    347
TRI5_5                              ARDAWEDFATGYISAHASIPRYRLQEVIGGQYIMDSASY.    322
TRI5_4                              ARDAWESFAKGYISSHFVNPRYRLRDVFGDQYLIDVASY.    181
TRI5_3                              AREAWESFAKGWISYHTSSARYRLRELIECQYITIDTC..    309
TRI5_1                              ARDVWDTFIKGYVYFHIISTRYRLHEILGDQYRWDIDHPT    336
Sparassis_crispa_XP_027613108.1     ARDAWENFVRGYTSFHISSPRYRLQEVVGSRYLID.....    330
Beauveria_bassiana_XP_008596951.    LLYIYRDFVMGYIEWHFRINRYKLDEIGLYLQE.......    320
Trichoderma_reesei_XP_006964535.    ILGAYADFMLGYMEWHFQVERYRLNELGLTLKS.......    306
Trametes_versicolor_XP_008037460.   LAKICRSYVVGYVEFHFRARRYRLEDLEMEY.........    317
Consensus                                     g    h    ry l

TRI5_2                              .......                                     334
TRI5_6                              .......                                     347
TRI5_5                              .......                                     322
TRI5_4                              .......                                     181
TRI5_3                              .......                                     309
TRI5_1                              VPKYANS                                     343
Sparassis_crispa_XP_027613108.1     .......                                     330
Beauveria_bassiana_XP_008596951.    .......                                     320
Trichoderma_reesei_XP_006964535.    .......                                     306
Trametes_versicolor_XP_008037460.   .......                                     317
Consensus
```

Figure S4.

```
scaffold2.t835                                                    ................MTVVNNAVTAEAADHFILPDLVSH     24
scaffold6.t228                                                    ........................MSATSGHFILPDLLSV     16
scaffold6.t230                                                    ........................MSSATKQFTLPDLLAM     16
scaffold6.t380                                                    ..............................MIKFPDLIGP     10
scaffold9.t175                                                    ........................................      0
XP_007772164.1_terpenoid_synthase_[Coniophora_puteana_RWD-64-59   .......................MGLPQATEFVLPDFFAP     17
XP_007771895.1_terpenoid_synthase_[Coniophora_puteana_RWD-64-59   .......................MSTMDPSEFILPDFFAT     17
QEP49715.1_alpha-muurolene_synthase_[Inonotus_obliquus]           MVKASGSSPASLLPLPTPTTVRSSTNQSPEGYVLPDLVSH     40
Consensus

scaffold2.t835                                                    CNFP.LKRHPNGDSIAASSDKWLDTGCPELTPENRAAIYG     63
scaffold6.t228                                                    LPFK.GSFNPHYTEAAAASSAWVN.QYRIVSDRKRAFFLQ     54
scaffold6.t230                                                    CPFT.GSTNPHYAKAAAESSAWVN.SYNILSDRKRAFFVT     54
scaffold6.t380                                                    IPFP.LRLNPHTRFASADSDAFMD.EMANFTEKQRTRFFG     48
scaffold9.t175                                                    ........................................      0
XP_007772164.1_terpenoid_synthase_[Coniophora_puteana_RWD-64-59   CPFTLGLTNPHADTVFPEARAWIG.KFLPFSG........     48
XP_007771895.1_terpenoid_synthase_[Coniophora_puteana_RWD-64-59   CPFAFGRTNPHADVVIPEAHAWIV.KHVPFVDRKRDEFIQ     56
QEP49715.1_alpha-muurolene_synthase_[Inonotus_obliquus]           CKFP.LSYNPHGDTVAVEADRWLDHGCPELTPKMRKALYG     79
Consensus

scaffold2.t835                                                    LKAGILTAHCYPDCDDEHLRVVADFLGYLFHLDNISDGMM    103
scaffold6.t228                                                    GGSELLCAHAYPYAGYEQLRTCCDLVNILFTVDEISDEQN     94
scaffold6.t230                                                    GSNELLVSHTYPYAGYEQFRTCCDFVNLLFVVDEVSDDQN     94
scaffold6.t380                                                    LNAGLLCGMCYAECGPEALRVCTDFMSFLFNLDDWSDEFD     88
scaffold9.t175                                                    ......................................MN      2
XP_007772164.1_terpenoid_synthase_[Coniophora_puteana_RWD-64-59   .....LAWHCDPWAGKEGFRTICDFQNLLFLMDELTDEMS     83
XP_007771895.1_terpenoid_synthase_[Coniophora_puteana_RWD-64-59   DGFQDLMPHCYPWAGKETLRTMCYLNNLLFLVDDLTDDMN     96
QEP49715.1_alpha-muurolene_synthase_[Inonotus_obliquus]           LHAGELTAFCYTTCDAHHLRVISDFMNYLFHLDNISDGMM    119
Consensus

scaffold2.t835                                                    SRGTRELADVVMNAHWFPDRYVPVHNTANTELIEEPSAGK    143
scaffold6.t228                                                    GKDASATGSILLNALRDPDFDDGSALAK....MVKEFKAQ    130
scaffold6.t230                                                    GQDARQTGNVYLNAMRDPAWDDGSALAK....MTKEFRAR    130
scaffold6.t380                                                    TTGTRGLEEVVMNTLRHPDTYHSDTVP.......AKITKS    121
scaffold9.t175                                                    DQGTLGTAVQVLNVLYHPNQSRPATR.......VGRMTKD     35
XP_007772164.1_terpenoid_synthase_[Coniophora_puteana_RWD-64-59   GEDARVVGESFIRVLKDPSVDNESIIAQ....ATREFRTR    119
XP_007771895.1_terpenoid_synthase_[Coniophora_puteana_RWD-64-59   SDEARGFGESFIRVLNDPAVHDSSQVMQ....ATREFRSR    132
QEP49715.1_alpha-muurolene_synthase_[Inonotus_obliquus]           TRDADLLSDVVMNALWFPHEHRPCKGQPAEEISAGKLARD    159
Consensus                                                                          p

scaffold2.t835                                                    LAR...EDAKPGPQARFKEHMELFFDAVQKQTEDRDAGII    180
scaffold6.t228                                                    LIK....FSVPACYRRFVKHCEDYVNALAVEAQLRERGEV    166
scaffold6.t230                                                    LLR....YAGPGCYRRFLKHCEDYVDAVAREAEYRERGYV    166
scaffold6.t380                                                    WWTRMLKTVGPRCQQRFIDTMGLYFEAIMQQAADRAQKNV    161
scaffold9.t175                                                    YWLRLIATGSSGAQQRFIETLDMFFQAVTQQAIDRANGVI     75
XP_007772164.1_terpenoid_synthase_[Coniophora_puteana_RWD-64-59   IADD..VATKSVWFGRFVAICKSYADATYIEAEHRENNRV    157
XP_007771895.1_terpenoid_synthase_[Coniophora_puteana_RWD-64-59   ITGT..GVTESRWFGRFLAIFKLYINAVCAEAEDRENKRI    170
QEP49715.1_alpha-muurolene_synthase_[Inonotus_obliquus]           YWLRCITDAGPDVQARFKENLQLFFEAVHIQSIHREHGEI    199
Consensus                                                                        rf         a       r

scaffold2.t835                                                    PDLESYIDMRRDTSGCKPSFDLIEYAMGIDLPDFVMDHPV    220
scaffold6.t228                                                    LDLDSYTVLRRENSAVRFCFGLFGFVLGHDLPDDIFEHPV    206
scaffold6.t230                                                    LDMASFETLRRENSAIRLCFGLFEYVLGVDLPDGVFEDPV    206
scaffold6.t380                                                    PELEAYITLRRNTSGCKTGFALIEYAAGIDLPNEVVEHPI    201
scaffold9.t175                                                    ADLESYIAMRRDTSGCKPCWALIEYANNLDLSWEVMDHPV    115
XP_007772164.1_terpenoid_synthase_[Coniophora_puteana_RWD-64-59   LDLDDFVIARRENSAVRCCFSINEHALGIDLPDSVFEDPE    197
XP_007771895.1_terpenoid_synthase_[Coniophora_puteana_RWD-64-59   LDLDTFTVARRENSAVMVFFAITEYALGIDLPDAVYEDPT    210
QEP49715.1_alpha-muurolene_synthase_[Inonotus_obliquus]           PDLEVYIDVRRDESGCKPVFDLIEYGLGINLPDFVIEHPI    239
Consensus                                                                  rr  s                l       p

scaffold2.t835                                                    IRALNQFANDLVTFVQLTSFSKAGLTRFLSVRWSNDVFSY    260
scaffold6.t228                                                    FMRMHLAAVDMVC...................WSNDIYSY    227
scaffold6.t230                                                    FMTLYWAAADMVC...................WSNDVYSY    227
scaffold6.t380                                                    IQNLLDATNDCVS...................WANDILSY    222
scaffold9.t175                                                    IRGLGEAANDLVT...................WSNDIFSY    136
XP_007772164.1_terpenoid_synthase_[Coniophora_puteana_RWD-64-59   FLRMYFDAVDMIV...................IVNDVYSY    218
XP_007771895.1_terpenoid_synthase_[Coniophora_puteana_RWD-64-59   FLRVYADSADMVI...................LVNDVFSY    231
QEP49715.1_alpha-muurolene_synthase_[Inonotus_obliquus]           IKALNQGSNDLVT...................WSNDIFSY    260
Consensus                                                                  d                        nd  sy

scaffold2.t835                                                    SVEQARGDT.HNMIVILMKHHGLSLQGAVNYVGELCKQTI    299
scaffold6.t228                                                    NMEQAMGHTGNNVVTVLMKVKGLDLQGAAEHVGVHFKNLM    267
scaffold6.t230                                                    NMEQAKGHSGNNIVTVLMRQKNVDLQTASDLVGEHFATLM    267
scaffold6.t380                                                    NREQSRGDS.HNLVPVIMKTIGLDRQPAIFYAGDLCNKSV    261
scaffold9.t175                                                    NVEQSKGDT.HNMIVVVQKQQDLDLQSAVNFVGDLCKQSI    175
XP_007772164.1_terpenoid_synthase_[Coniophora_puteana_RWD-64-59   NMEQAKGLAGNNVVTVLEQALGVDLQAAVNRGGEMFAQKM    258
XP_007771895.1_terpenoid_synthase_[Coniophora_puteana_RWD-64-59   NREQAKGLDGNNNITVLMQTLDLDLQAAVDHVGEMFSQKM    271
QEP49715.1_alpha-muurolene_synthase_[Inonotus_obliquus]           NVEQARGET.HNMIIILEQRYGMELQDAMDYVGEMCRVTM    299
Consensus                                                           eq  g    n             q a    g

scaffold2.t835                                                    DAFVENQQSIPSWG.PEIDTDVTVYVKGLQDWIVGSLHWS    338
scaffold6.t228                                                    DSFVADKARLPSWG.PEWDPVVADFVMAMENWLIGNCEWS    306
scaffold6.t230                                                    DRFVTAKGGLPSWS.PSVDAAVSDYVRAMEYWVTGNLEWS    306
scaffold6.t380                                                    ERFLTSKAMLPSWG.SEVDTQVNMYVQGLEDWMIANGEWS    300
scaffold9.t175                                                    DRFSVLRENLPSWG.PEIDRQVELYVDGLADWITGSLKWS    214
XP_007772164.1_terpenoid_synthase_[Coniophora_puteana_RWD-64-59   EGYVRGRGVPPSWG.AKVDADVEHFFDSVDQWIVGNLEWS    297
XP_007771895.1_terpenoid_synthase_[Coniophora_puteana_RWD-64-59   EGCMRGRAMLPSWG.VKVDADVERFFDALDQWVVGNLEWS    310
QEP49715.1_alpha-muurolene_synthase_[Inonotus_obliquus]           ENFVANKARIPSFGDAQLDRDVAGYVQGLQDWIVGALHWS    339
Consensus                                                                   ps      d  v         w      ws

scaffold2.t835                                                    FVTERYFGRDGAKVKKYRVVKLLSKVNKNDA.........    369
scaffold6.t228                                                    FETQRYFGPERAEVKRTRVVRLYPKREQEDD.........    337
scaffold6.t230                                                    FETQRYFGVMHAEIKYTRLISLREREEEE...........    335
scaffold6.t380                                                    FMTERYFGKDGPKIKKSMRVPLLPQLGFD...........    329
scaffold9.t175                                                    FESERYFGKTGLEVKKTRVVALLPRRA.............    241
XP_007772164.1_terpenoid_synthase_[Coniophora_puteana_RWD-64-59   TETSRYLGPDHEEIMKTGRVVLRKIESKTK..........    327
XP_007771895.1_terpenoid_synthase_[Coniophora_puteana_RWD-64-59   SQSPRYLGPEHEEIMRTRRVVLRKVETEIE..........    340
QEP49715.1_alpha-muurolene_synthase_[Inonotus_obliquus]           FMSQRYFGTEGAEVKKHRYVKLLPKKESTTSTAGTERKSP    379
Consensus                                                             ry g             l

scaffold2.t835                                                    ........................................    369
scaffold6.t228                                                    ........................................    337
scaffold6.t230                                                    ........................................    335
scaffold6.t380                                                    ........................................    329
scaffold9.t175                                                    ........................................    241
XP_007772164.1_terpenoid_synthase_[Coniophora_puteana_RWD-64-59   ........................................    327
XP_007771895.1_terpenoid_synthase_[Coniophora_puteana_RWD-64-59   ........................................    340
QEP49715.1_alpha-muurolene_synthase_[Inonotus_obliquus]           ECLSTNRSDAQSAYLPLRLIYRAGRGMRNMASWLLQFFNH    419
Consensus
```

Figure S5.

```
Polyprenyl_synthetase861                                          MLNLSNLRSLLSQVLSLPALHTVVLFTPEGQLVSFAADVY     40
KAI0652351.1_isoprenoid_synthase_domain-containing_protein_[Tra   MLNLANLRTLLSRVLAPPSLHTVALFTPEGELVCYASDPS     40
KAI0673618.1_isoprenoid_synthase_domain-containing_protein_[Tra   MLNLANLRTLLSRVLAPPSLHTVALFTPEGELVCYASDPS     40
Consensus                                                         mlnl nlr lls vl  p lhtv lftpeg lv  a d

Polyprenyl_synthetase861                                          RSKDNVRVVVGLSSEVWQETKEQGMGMVDSELGRVLVLPV     80
KAI0652351.1_isoprenoid_synthase_domain-containing_protein_[Tra   KTKDHVRVLVGLSTETWQETREQHVGRVDSELGHLLVLPI     80
KAI0673618.1_isoprenoid_synthase_domain-containing_protein_[Tra   KTKDHVRVLVGLSTETWQETREQHVGRVDSELGHLLVLPI     80
Consensus                                                           kd vrv vgls e wqet eq  g vdselg  lvlp

Polyprenyl_synthetase861                                          EPVRKVHEERVDEPLMLLALNAEDSISWSELENKARELAK    120
KAI0652351.1_isoprenoid_synthase_domain-containing_protein_[Tra   ESPKKEDDD...EPLLLLALNSEASVPWEQLEAKARKLAE    117
KAI0673618.1_isoprenoid_synthase_domain-containing_protein_[Tra   ESPKKEDDD...EPLMLLALNSEASVPWEQLEAKQKKASP    117
Consensus                                                         e   k       epl llaln e s  w  le k

Polyprenyl_synthetase861                                          HLAEPVLELRGRLST..GPILLISPR............AE    146
KAI0652351.1_isoprenoid_synthase_domain-containing_protein_[Tra   HLEKPVSQLSQTLAAPVAPQKKASPAQPQVPPTSDTFLTT    157
KAI0673618.1_isoprenoid_synthase_domain-containing_protein_[Tra   AQPQAPPTSDTFLTT.........................    132
Consensus                                                                     l

Polyprenyl_synthetase861                                          PPPPKPPVRSDPYSLLGPQLERLRETLLHLLGSSHPGLSE    186
KAI0652351.1_isoprenoid_synthase_domain-containing_protein_[Tra   PNPAKPPARPDPYALVAPQLKELRETLLNLLGSSHPSLTE    197
KAI0673618.1_isoprenoid_synthase_domain-containing_protein_[Tra   SSPAKPPARPDPYALVAPQLKELRETLLNLLGSSHPSLTE    172
Consensus                                                           p kpp r dpy l  pql  lretll llgsshp l e

Polyprenyl_synthetase861                                          IAKYYFLHPSKQLRPLLVLLFAQATNGLGNGWHLKQWAAE    226
KAI0652351.1_isoprenoid_synthase_domain-containing_protein_[Tra   IAKYYFLHPSKQLRPLLVLLFSQATNGLGSGWYLKQWAAE    237
KAI0673618.1_isoprenoid_synthase_domain-containing_protein_[Tra   IAKYYFLHPSKQLRPLLVLLFSQATNGLGSGWYLKQWAAE    212
Consensus                                                         iakyyflhpskqlrpllvllf qatnglg gw lkqwaae

Polyprenyl_synthetase861                                          CEGAGGRAEELDRPLTRADVLNDWNPNMPDNTASFESPFS    266
KAI0652351.1_isoprenoid_synthase_domain-containing_protein_[Tra   CEGAGGRAEELDWPLTRADVLNDWNPSMPDHTASFQSSFS    277
KAI0673618.1_isoprenoid_synthase_domain-containing_protein_[Tra   CEGAGGRAEELDWPLTRADVLNDWNPSMPDHTASFQASFS    252
Consensus                                                         cegaggraeeld pltradvlndwnp mpd tasf   fs

Polyprenyl_synthetase861                                          LRPPRPPVQPPLPPPFPPAHDTLTLSIPPCLLPTQIRLAQ    306
KAI0652351.1_isoprenoid_synthase_domain-containing_protein_[Tra   LRSPRAPHKPTLPPPFPPALHVPTVAAPLTLLPTQVRLAQ    317
KAI0673618.1_isoprenoid_synthase_domain-containing_protein_[Tra   LRSPRAPHKPPLPPPFPPALHAPTVAAPLALLPTQVRLAQ    292
Consensus                                                         lr pr p  p lpppfppa    t   p  llptq rlaq

Polyprenyl_synthetase861                                          IVEMIHVASLLHDDVIDKSPLRRGVPSAPAAFGNKLTILG    346
KAI0652351.1_isoprenoid_synthase_domain-containing_protein_[Tra   IVEMIHVASLLHDDVIDKSPLRRGAASAPAAFGNKLSVLA    357
KAI0673618.1_isoprenoid_synthase_domain-containing_protein_[Tra   IVEMIHVASLLHDDVIDKSPLRRGAASAPAAFGNKLSVLA    332
Consensus                                                         ivemihvasllhddvidksplrrg  sapaafgnkl  l

Polyprenyl_synthetase861                                          GDFLLGRASATLSRLGDNEVVELIASVIANLVEGEILQLK    386
KAI0652351.1_isoprenoid_synthase_domain-containing_protein_[Tra   GDFLLGRTSAALSRLGENEVVELIASVIANLVEGEILQLK    397
KAI0673618.1_isoprenoid_synthase_domain-containing_protein_[Tra   GDFLLGRTSAALSRLGENEVVELIASVIANLVEGEILQLK    372
Consensus                                                         gdfllgr sa lsrlg nevveliasvianlvegeilqlk

Polyprenyl_synthetase861                                          SVHGEELGIAG.APTLGKDYFNIYLQKTYMKTASLMAKGA    425
KAI0652351.1_isoprenoid_synthase_domain-containing_protein_[Tra   SVHADELGLAGLSPNVGQENWNIYLQKTYLKTASLMAKGA    437
KAI0673618.1_isoprenoid_synthase_domain-containing_protein_[Tra   SVHADELGLAGLSPNVGQENWNIYLQKTYLKTASLMAKGA    412
Consensus                                                         svh  elg ag  p  g    niylqkty ktaslmakga

Polyprenyl_synthetase861                                          RAAVVLGGCKEGEVWKEVAYAYGRNLGIAFQLVDDILDYE    465
KAI0652351.1_isoprenoid_synthase_domain-containing_protein_[Tra   RAAVVLGGCKEGEVWKEVAYAYGRNLGIAFQLVDDILDYE    477
KAI0673618.1_isoprenoid_synthase_domain-containing_protein_[Tra   RAAVVLGGCKEGEVWKEVAYAYGRNLGIAFQLVDDILDYE    452
Consensus                                                         raavvlggckegevwkevayaygrnlgiafqlvddildye

Polyprenyl_synthetase861                                          AGEATLGKPGGADLQLGLATGPALFAWEEHPEMGPLIKRK    505
KAI0652351.1_isoprenoid_synthase_domain-containing_protein_[Tra   AGDATLGKPGGADLQLGLATGPALFAWEEHPELGPLIKRK    517
KAI0673618.1_isoprenoid_synthase_domain-containing_protein_[Tra   AGDATLGKPGGADLQLGLATGPALFAWEEHPELGPLIKRK    492
Consensus                                                         ag atlgkpggadlqlglatgpalfaweehpe gplikrk

Polyprenyl_synthetase861                                          FEREGDVELVSALS....GPYRRRH...............    526
KAI0652351.1_isoprenoid_synthase_domain-containing_protein_[Tra   FGQPGDVELARDLVRRSSGVERTRDLARAHADKAREVLAV    557
KAI0673618.1_isoprenoid_synthase_domain-containing_protein_[Tra   FGQPGDVELARDLVRRSSGVERTRDLARAHADKAREVLAV    532
Consensus                                                         f   gdvel   l     g  r r

Polyprenyl_synthetase861                                          .....................                       526
KAI0652351.1_isoprenoid_synthase_domain-containing_protein_[Tra   LPDSDAKIALEVLTERVVKRT                       578
KAI0673618.1_isoprenoid_synthase_domain-containing_protein_[Tra   LPDSDAKVALEVLTERVVKRT                       553
Consensus
```

Figure S6.

```
scaffold13.t100                                                   ........................................    0
OBZ72750.1_Protein_farnesyltransferase_subunit_beta_[Grifola_fr   MTERHLRSTPTDGYATPTSHIQAATEQILRSHLPS.....   35
XP_027616833.1_Protein_farnesyltransferase_subunit_beta_[Sparas   ..........MDGYPTATSSLQSVTENVLKAHLPSSETYQ   30
Consensus

scaffold13.t100                                                   ........................................    0
OBZ72750.1_Protein_farnesyltransferase_subunit_beta_[Grifola_fr   ...........ASEANQGPVLQKNIHLQYLLRNLVQGFPE   64
XP_027616833.1_Protein_farnesyltransferase_subunit_beta_[Sparas   AAGATGDSSGKAGTSETGPLLQKQLHIQFLLRNLLQGFPE   70
Consensus

scaffold13.t100                                                   ........................................    0
OBZ72750.1_Protein_farnesyltransferase_subunit_beta_[Grifola_fr   RYTSQDASQPWLIFWTLQGFSVLGVGLDDQTKKRAIETLL  104
XP_027616833.1_Protein_farnesyltransferase_subunit_beta_[Sparas   RYISQDASQPWLIYWTLQGFSVLGIGMDDQTRKRAKQTLL  110
Consensus

scaffold13.t100                                                   ........................................    0
OBZ72750.1_Protein_farnesyltransferase_subunit_beta_[Grifola_fr   ALQHPEGGFGGGPGQAAHLLPTYAAVCALAIVGRPGEGGG  144
XP_027616833.1_Protein_farnesyltransferase_subunit_beta_[Sparas   ALQHPYGGFSGGPGQAAHLLPTYAAVCALAIVGQPGPDGA  150
Consensus

scaffold13.t100                                                   ........MYDFFMSLKQPDGSFLVTHHGEVDVRGLYCLI   32
OBZ72750.1_Protein_farnesyltransferase_subunit_beta_[Grifola_fr   WEVIDRQKMYKFFMSLKQQDGSFLVMHHGEVDMRGIYCLI  184
XP_027616833.1_Protein_farnesyltransferase_subunit_beta_[Sparas   WDEIDRTKMYEFFLSLKQPDGSYTVTQDGEVDVRGLYCLL  190
Consensus                                                                 my ff slkq dgs  v   gevd rg ycl

scaffold13.t100                                                   AVATLLNIATPQLLAGMPEFIASCQTYEGGFGNASFPEWA   72
OBZ72750.1_Protein_farnesyltransferase_subunit_beta_[Grifola_fr   TVATMLNMLTPELLAGVPEFIASCQTYEGGFGNASFPGWV  224
XP_027616833.1_Protein_farnesyltransferase_subunit_beta_[Sparas   STATLLNIVTPELLKGMPEFIVSCQSYEGGFGNASFPEWA  230
Consensus                                                           at ln  tp ll g pefi scq yeggfgnasfp w

scaffold13.t100                                                   FQ......DNGNGVKFNPSVLRPVLGEAHGGYTFCAIATW  106
OBZ72750.1_Protein_farnesyltransferase_subunit_beta_[Grifola_fr   FEEDDPERDGSSTDSFNPSSPRPPLGEAHGGYTFCATATW  264
XP_027616833.1_Protein_farnesyltransferase_subunit_beta_[Sparas   FP.......EEDDGAFDASAPRPLLGEAHGGYTFCATATW  263
Consensus                                                         f              f  s  rp lgeahggytfca atw

scaffold13.t100                                                   VLLQPYLRLYYPASPSSASVAGSSASHAPSINLHTLMRWL  146
OBZ72750.1_Protein_farnesyltransferase_subunit_beta_[Grifola_fr   VLLQPYLRLYYPSS.............APAINRSALLRWL  291
XP_027616833.1_Protein_farnesyltransferase_subunit_beta_[Sparas   VLLQPYLRHYYSSS.........STSQRPTVNMHALLRWL  294
Consensus                                                         vllqpylr yy  s              p  n   l rwl

scaffold13.t100                                                   TQMQGTPIELGGFRGRTNKLVDGCYSWWVGVAVVLVEGLL  186
OBZ72750.1_Protein_farnesyltransferase_subunit_beta_[Grifola_fr   TQMQGIPAEIGGFRGRTNKLVDGCYSWWVGGCVVLAETLL  331
XP_027616833.1_Protein_farnesyltransferase_subunit_beta_[Sparas   TQMQGTHMELGGFRGRTNKLVDGCYSWWVGGCVGLVEGLL  334
Consensus                                                         tqmqg   e ggfrgrtnklvdgcyswwvg  v l e ll

scaffold13.t100                                                   ETGTSAG..SSAEGRAAATHQEDAWDDVDDSLFNRHALQE  224
OBZ72750.1_Protein_farnesyltransferase_subunit_beta_[Grifola_fr   GIGLGGDRRPEERGKGEEHAGEDGWDDVDDSLFDRRALQE  371
XP_027616833.1_Protein_farnesyltransferase_subunit_beta_[Sparas   GPGCVPG..AHEPG.....AHAEEWTDIDDSLFNRQALQE  367
Consensus                                                           g          g          w d ddslf r alqe

scaffold13.t100                                                   YILYAGQHPAGGLRDKPPK.....................  243
OBZ72750.1_Protein_farnesyltransferase_subunit_beta_[Grifola_fr   YILYAGQHPAGGLRDKPPKPSDSYHTLYCLSGLSAAQHRV  411
XP_027616833.1_Protein_farnesyltransferase_subunit_beta_[Sparas   YILYAGQYPAGGLRDKPPKSADSYHTLYCLSGLSAAQHTV  407
Consensus                                                         yilyagq pagglrdkppk

scaffold13.t100                                                   ........................................  243
OBZ72750.1_Protein_farnesyltransferase_subunit_beta_[Grifola_fr   IPHDARRAEVLASWRGDIAKEKKCAGLAAPSGDG.EILEN  450
XP_027616833.1_Protein_farnesyltransferase_subunit_beta_[Sparas   LPDDARRAELLASWSDETINEREGFGFTPASDAARSQWDA  447
Consensus

scaffold13.t100                                                   ........................................  243
OBZ72750.1_Protein_farnesyltransferase_subunit_beta_[Grifola_fr   LRREIFLDTLSWSEEEGTVKYVGGSGNRVNATHPLFNLTV  490
XP_027616833.1_Protein_farnesyltransferase_subunit_beta_[Sparas   MRKEAFVSALSWVEEEGTSKYVGGAVNRVNATHPLFNLTA  487
Consensus

scaffold13.t100                                                   ..............................            243
OBZ72750.1_Protein_farnesyltransferase_subunit_beta_[Grifola_fr   THSEAIMAYFYGQTLPVRKPHHAASTPPTG            520
XP_027616833.1_Protein_farnesyltransferase_subunit_beta_[Sparas   THTEGMMQHFYGQQVPERRPKHPAAEK...            514
Consensus
```

Figure S7.

```
scaffold8.t181              MGALSMLVLLLTHPLEFRTLLQYKLWHESNRDITAPSELE     40
Lentinula_edodes_GAW09328   MGATAWLTLLLTHPLEFRTLLQFWLFHEQKRDITSMKEHA     40
Consensus                   mga   l lllthplefrtllq  l he  rdit   e

scaffold8.t181              NSGWNRKSMRRCWELLDMTSRSFAGVIKELEGDLARIICL     80
Lentinula_edodes_GAW09328   TSGWDRQTMRRCWEFLDMTSRSFSAVIKEVEGDLARTICM     80
Consensus                    sgw r  mrrcwe ldmtsrsf  vike egdlar ic

scaffold8.t181              FYLVLRGLDTIEDDMTLPDEKKQPLLRSFHELTVTPGWTF    120
Lentinula_edodes_GAW09328   FYLVLRGLDTIEDDMTIPDDVKQPILRSFHKLTITPGWTY    120
Consensus                   fylvlrgldtieddmt pd  kqp lrsfh lt tpgwt

scaffold8.t181              NGSGPYEKDRQLLVEYAVVSEELNRVDTKYLDVIIDITQK    160
Lentinula_edodes_GAW09328   NDSGPTEKDRQLLVEYDKVIEEVNLLDPEYKSIILDITEK    160
Consensus                   n sgp ekdrqllvey  v ee n  d  y   i dit k

scaffold8.t181              MENGMADYAHRAATTGEVYVEKISDYDLYCHYVAGLVGEG    200
Lentinula_edodes_GAW09328   MENGMADFAHRAATAGATYIDKVEDYDLYCHYVAGLVGEG    200
Consensus                   mengmad ahraat g  y  k  dydlychyvaglvgeg

scaffold8.t181              LTRLWSASGKEAPWLGEQLELANSMGLMLQKTNIIRDYRE    240
Lentinula_edodes_GAW09328   LSRLFSASKKEVSWLGTQLEISNSMGLLLQKTNIIRDYRE    240
Consensus                   l rl sas ke  wlg qle  nsmgl lqktniirdyre

scaffold8.t181              DVEDRRFFWPREIWGREVYGAACGRPAFTKMEQMYQPGSE    280
Lentinula_edodes_GAW09328   DCDDKRYFWPKEIWGKKEYG.......FQEMKDMYLPGAE    273
Consensus                   d  d r fwp eiwg   yg       f  m   my pg e

scaffold8.t181              KQALWVLSGMVVDVLGHAVDSLDYLRLLRRQSVFNFCAIP    320
Lentinula_edodes_GAW09328   ERAQWVQSEMILDAMRHLTDALDYLRLLKNQSVFTFCAIP    313
Consensus                     a wv s m  d   h  d ldylrll  qsvf fcaip

scaffold8.t181              QTMAMATLQLCFMNYEMFQRNIKIRKAEAASLIMRSTNPR    360
Lentinula_edodes_GAW09328   ATMAIATLDLCFMNPAMFQRNVKIRKAEAASLIMRSVNPR    353
Consensus                    tma atl lcfmn  mfqrn kirkaeaaslimrs npr

scaffold8.t181              DVAYIFRDYARKIHSKAVPEDPSFLQISVACGKIEQWCEH    400
Lentinula_edodes_GAW09328   DVAEIFRHHCRSIHAKAVPEDPNFLRISVACGKVEQWCEH    393
Consensus                   dva ifr   r ih kavpedp fl isvacgk eqwceh

scaffold8.t181              HYPSFVSVLHSPASGNTQQVFDKSDARTRIMEASEKRDRE    440
Lentinula_edodes_GAW09328   NFPSFV...RMPSTAGNPPTFDPEDARTSILKKSQEIDQT    430
Consensus                     psfv     p        fd  dart i   s   d

scaffold8.t181              LQLRKRAKELSANGKMNGVNSSAHQLQEQGPSTKEIIMYI    480
Lentinula_edodes_GAW09328   ITQRKRVAQLQEK...QGSNGEAKPALAQQGVPWELFAVV    467
Consensus                      rkr   l       g n  a     q     e

scaffold8.t181              AAAFIIVFAVGLGGFWLLLKYFG.                    503
Lentinula_edodes_GAW09328   GAIFGILLISILGVVWIVVMFSDK                    491
Consensus                    a f i     lg  w
```
